# Navigating Professional Aspirations: The Role of Work–Family Conflict, Job Demand and Control, and Social Support in Shaping Nursing Students' Career Intentions

**DOI:** 10.1155/jonm/9974453

**Published:** 2025-10-31

**Authors:** Andrea Gilchrist, Denisa Večerková, Andrea Pokorná, Abanoub Riad

**Affiliations:** ^1^Department of Health Sciences, Faculty of Medicine, Masaryk University, Brno, Czech Republic; ^2^Department of Public Health, Faculty of Medicine, Masaryk University, Brno, Czech Republic; ^3^Masaryk Centre for Global Health (MCGH), Department of Public Health, Faculty of Medicine, Masaryk University, Brno, Czech Republic

**Keywords:** career choice, cross-sectional studies, nursing, occupational stress, research design, social support, statistical analysis, students, surveys and questionnaires, work-life balance

## Abstract

**Background:**

Nurses are the backbone of healthcare and vital to patient outcomes. Newly qualified nurses face significant challenges, with many leaving the profession within 2 years, worsening the nursing shortage.

**Methods:**

This study aimed to examine how work-to-family conflict, job demands, job control, and social support influence the career intentions of nursing students in the Czech Republic. A nationwide cross-sectional survey was conducted between August and December 2023 using a self-administered online questionnaire. The instrument was adapted from the Nurses Early Exit Study and included validated measures related to psychosocial working conditions and work-to-family balance. Data were analyzed using IBM SPSS Statistics to explore associations between these factors, health-related characteristics, and students' intentions to remain in or leave the nursing profession.

**Results:**

Among 559 respondents, WAI scores were significantly associated with gender, self-perceived health (*ρ* = 0.531), and exhaustion (*ρ* = −0.542). Social support improved training expectations alignment (AOR = 1.021, *p* < 0.001) and increased likelihood of pursuing nursing qualifications (AOR = 1.308, *p*=0.004). Conversely, exhaustion negatively influenced endurance (AOR = −0.247, *p*=0.030) and increased the likelihood of switching careers (AOR = 1.145, *p*=0.019). Work ability positively predicted endurance (AOR = 0.309, *p* < 0.001) and reduced career-shift intentions (AOR = 0.891, *p*=0.003). Despite strong training endurance, 55.6% considered leaving nursing, citing exhaustion and work–family conflict as key factors.

**Conclusion:**

Social support emerged as a protective factor, aligning training expectations with clinical experiences and encouraging further nursing qualifications. Work ability promoted endurance in training and reduced career shift. In contrast, exhaustion, work–family conflict, poor health, and negative clinical experiences—including bullying—undermined retention. Nursing education administrators and healthcare managers should prioritize supportive environments, strengthen mentorship and peer support, and implement interventions to reduce stress and improve clinical placements to enhance retention and workforce sustainability.

## 1. Introduction

Nurses, as the largest group of healthcare professionals, play a critical role in influencing patient care quality, healthcare outcomes, and population health [1, 2]. The increasing complexity of patient needs, shorter hospital stays, and the rapid growth of healthcare technology and specialization have amplified the demand for highly experienced nurses [3]. This demand is further driven by the aging population and the growing need for healthcare services [4, 5], which underscores the importance of appropriate staffing and skill mix as crucial determinants of healthcare performance and quality assurance in patient care [6]. Despite the recognition of the importance of a sustainable health workforce in achieving health and development goals, global challenges in workforce recruitment and retention persist [4, 6, 7].

The Job Demands, Control, and Support (JDCS) model, developed by Karasek [8], provides a comprehensive framework for understanding how job demands, job control, and social support interact to influence well-being, job satisfaction, and turnover intentions among nursing professionals and students [9, 10].

The intention to leave is a critical issue across Europe, with 9.5% of recently qualified nurses and 1 in 4 graduating nursing students indicating plans to leave the profession [2]. Multiple studies describe the first 2 years postgraduation as a critical period during which many new nurses decide to leave the profession, which not only worsens the ongoing nursing shortage but also increases healthcare costs and compromises patient care and safety [3, 11]. Maier et al. [12] reported that Generation Z nurses (born in 1997 and later) display the highest rates compared to older generations. The current workforce predominantly includes four generational groups: baby boomers (1946–1964), Generation X (1965–1980), millennials (1981–1996), and generation Z (mid-1990s to early 2010s) [3]. Generation Z nurses play an essential and central role in supporting and sustaining the nursing profession in the future; understanding their approach to emotional labor is needed, as research indicates they face significant challenges in emotional regulation and report higher rates of mental health concerns compared to previous generations [13]. Li et al. [13] described that Generation Z nurses (36%) are more likely to report emotional regulation difficulties compared to millennials (27%) and Generation X (20%), which aligns with findings from Bakker et al. [14], who reported high levels of depression, anxiety, distress, and burnout among nursing students and newly graduated staff, indicating a greater vulnerability to the emotional demands of nursing. Family structure was found to play a key role in emotional regulation, with extended families providing better emotional support and reducing depression risks [13].

Several studies have highlighted the significant impact of family-to-work conflicts (FWCs) on nursing professionals. For instance, a study by Navajas-Romero et al. [15] involving 991 nursing professionals across 35 European countries identified that physical demands were significantly associated with work-to-family work–life balance. Similarly, Wong et al. [16] found that both WFC and FWC were moderately correlated and that WFC showed moderate positive correlations with organizational turnover intentions and professional turnover intentions among nursing professionals. These conflicts have been linked to detrimental outcomes such as poor physical health, anxiety, depression, reduced life satisfaction, and job dissatisfaction [17].

While research predominantly focuses on all health professionals or concentrates on the nursing workforce as a whole, there is limited investigation on recruitment and retention, specifically among nursing students at various stages of their education [12]. It is important to understand that newly qualified registered nurses are essential to the future strength, stability, and effectiveness of the healthcare system. Studies have shown that they face significant challenges transitioning into their roles, often struggling with limited clinical experience and unfamiliar hospital protocols. In fact, many newly qualified nurses take over a year to gain confidence in their practice [2, 11]

By applying these established models to the context of nursing students, this study aims to offer valuable insights into the factors influencing their career intentions, endurance in training, and professional commitment. Specifically, this study seeks to answer the following research question: *How do work–family conflict, job demands, job control, and social support influence the career intentions of nursing students in the Czech Republic?*

## 2. Materials and Methods

### 2.1. Design

This analytical cross-sectional survey-based study was carried out in collaboration with representatives from the Czech Ministry of Health, educators, and various researchers in the fields of pedagogy and nursing education. Its objective was to identify factors affecting the retention and turnover of nursing students in the Czech Republic. The study was designed and reported in accordance with the Strengthening the Reporting of Observational Studies in Epidemiology (STROBE) guidelines for cross-sectional studies (see Supporting information: ‘STROBE Checklist' (available here)).

### 2.2. Settings

Nursing education in the Czech Republic is delivered through accredited undergraduate programmes that integrate theoretical instruction with practical clinical placements in hospitals and other healthcare facilities. These programmes are nationally regulated by the Ministry of Health to ensure consistent educational standards and to adequately prepare students for professional nursing roles. Since the educational reform in 2004, nursing education has transitioned away from secondary nursing schools to institutions of higher education. This transformation marked the end of professional nurse training at the secondary school level, shifting it to higher professional schools and universities, both of which may be university or nonuniversity in type. To qualify as a general nurse, students can choose between two educational pathways: studying at a higher professional nursing school (resulting in a diploma) or completing a bachelor's degree program at a university (resulting in a BSc. degree) [18].

Data for this study were collected using the JISC Online Surveys platform (accessed via the University of Oxford) through a self-administered questionnaire (SAQ). The data collection took place between August and December 2023.

### 2.3. Population

The study targeted nursing students enrolled in undergraduate nursing programmes in the Czech Republic who met the following criteria.

#### 2.3.1. Inclusion Criteria

• Enrollment in an undergraduate nursing program
• Fluency in the Czech language
• Aged 18 years or older
• Willingness to provide informed consent

#### 2.3.2. Exclusion Criteria

• Enrollment in non-nursing programmes
• Under 18 years of age
• Unwillingness to provide informed consent

Participants were recruited through a multichannel online strategy supported by the Czech Ministry of Health and university vice-deans. Recruitment involved email invitations distributed via university mailing lists, announcements on university portals, and personal nursing student contacts.

This approach aimed to maximize reach and ensure a representative sample. The required sample size of 589 was calculated using Epi Info™ based on an estimated target population of 32,000 healthcare students [19], with a 95% confidence level, 4% margin of error, and 50% outcome probability.

### 2.4. Instrument

The online questionnaire used in this study was adapted, with permission from the authors, from the Nurses Early Exit Study (NEXT Study), a European commission–supported project investigating factors contributing to nurses' early departure from the profession, involving 39,689 nurses across 10 European countries [20, 21]. Additional items were incorporated from Bakker et al. [22], who explored nursing students' clinical training experiences, academic expectations, dropout rates, and related protective and risk factors through a longitudinal study of three cohorts over 2.5 years.

#### 2.4.1. Sociodemographic and Health-Related Characteristics

The first part of the questionnaire assessed sociodemographic characteristics, such as gender, age, and body mass index (BMI); education-related attributes, including the type of secondary school attended, current nursing study program, current year of study, and study form; and work-related attributes, such as employment status, workload, and type of workplace. Moreover, health perceptions, including self-perceived health status (reflecting an individual's overall evaluation of their physical and mental health) and perceived exhaustion (indicating the extent of physical and emotional fatigue experienced), were measured using four 5-point Likert scale items each, yielding scores ranging from 4 to 20 for both measures. Anamnesis of musculoskeletal pain and sleep quality were also evaluated. Additionally, participants were asked about prior challenging experiences during clinical practice, such as exposure to patient mortality, human suffering, aggressive or problematic patients, and instances of bullying or discrimination.

#### 2.4.2. JDCS Model

The JDCS components were assessed using Likert scale items with composite scores of 4–16 for job demand, 6–24 for job control, and 5–23 for social support. Job demand demonstrated strong validity (CFI = 0.985, TLI = 0.954, and RMSEA = 0.066), while job control and social support showed acceptable reliability (social support: Cronbach's *α* = 0.810 and McDonald's *ω* = 0.814).

#### 2.4.3. Work Ability Index (WAI)

The WAI, a previously validated and widely used tool, assessed work ability in relation to past capacity and current study or job demands, covering current illnesses, health-related work impacts, absenteeism, and projected work ability over the next two years, with scores ranging from 7 to 49 [23].

#### 2.4.4. Work and Family Conflict Scale (WAFCS)

The WAFCS assesses the bidirectional conflict between work and family roles. It comprises two subscales: work-to-family conflict (WFC) and FWC, each measured using five items rated on a 7-point Likert scale ranging from 1 (*very strongly disagree*) to 7 (*very strongly agree*). Composite scores for both WFC and FWC range from 5 to 35, with higher scores indicating greater conflict [24].

#### 2.4.5. Training Feedback and Career Intentions

This section focused on the key outcome variables.

• *Training Expectations Alignment* (TEA) was assessed using two items on a 10-point Likert scale, evaluating whether clinical practice met students' expectations of working in healthcare and complemented their studies, with a composite score ranging from 2 to 20.
• *Endurance in Nursing Training* (ENT) included three items—expectations to stay in healthcare after graduation, successfully completing the current program, and considering dropping out (reverse scored)—rated on a 10-point Likert scale, with composite scores ranging from 3 to 30.
• *Career Intentions* covered plans to increase qualifications in nursing or outside nursing, quit the nursing profession, or start a different qualification, assessed through Yes/No responses and 5-point Likert scales (*Never to Daily*) for frequency.

### 2.5. Ethics

The study protocol was rigorously reviewed and approved by the Institutional Ethical Review Board (ERB) of xxxxxx anonymized for review on June 22, 2023 (approval reference: EC no. MU-IS/141052/2023/2054036/LF). All research procedures were conducted in strict accordance with the ethical principles outlined in the Declaration of Helsinki for research involving human participants, ensuring the protection of human rights, dignity, and well-being. Furthermore, full compliance with the European Union General Data Protection Regulation (GDPR) was maintained to safeguard the privacy and confidentiality of participant data throughout the study. Participants provided digital informed consent before participating in the study and could withdraw at any time without any consequences.

### 2.6. Analyses

Statistical analyses were conducted using IBM SPSS Statistics, Version 28, and Jamovi (R-based software), Version 2.3. The Shapiro–Wilk test was applied to evaluate the normality of numerical variables, for example, JDCS, WAI, and WAFCS scores, with statistical significance defined as *p* < 0.05. Descriptive statistics were presented as medians and interquartile ranges (IQRs) for numerical data, while categorical variables were summarized using frequencies (*n*) and percentages (%).

To examine associations, a range of inferential tests was employed, including the chi-squared test (*χ*^2^), Fisher's exact test, Mann–Whitney test (*U*), Kruskal–Wallis test (*H*), and Spearman's rho correlation, focusing on the relationships between independent variables (e.g., sociodemographic and health-related characteristics) and dependent variables (e.g., JDCS, WAI, and WAFCS scores). Furthermore, regression analyses were performed to identify important factors influencing training feedback and future career intentions. All statistical tests were carried out with a significance threshold of *p* < 0.05.

## 3. Results

### 3.1. Sample Characteristics

A total of 594 questionnaires were collected, and after applying the inclusion criteria, 559 valid responses were analyzed. The sample consisted predominantly of female (91.9%) nursing students, with the majority aged ≤ 21 years (58.4%).

In terms of education, nearly all students were enrolled in bachelor's degree programmes (97.3%), with the largest proportion in their third year (38.5%). Full-time study was more common (71.9%) compared to part time. The majority of participants (71.7%) had attended healthcare-specialized secondary schools, while only 17.7% and 10.6% attended general secondary schools and nonhealthcare-specialized secondary schools, respectively.

Employment was reported by 53.3% of students, with most engaged in part-time work (42.1% working ≤ 20 h per week and 21.8% for > 20 h per week) and 36% working full time. Among employed students, 62.5% worked in standard care departments, 21.8% in intensive care units, and 15.7% in other healthcare settings (Table 1).

### 3.2. JDCS

Overall, gender, age, and BMI had no significant impact on any of the JDCS constructs. Moreover, education- and employment-related attributes had no considerable association with JDCS constructs. The median job demand score was 12 (IQR: 10–13). Higher job demand score was significantly associated with attendance at healthcare-specialized secondary schools (*p* < 0.001). Self-perceived health (*ρ* = −0.252, *p* < 0.001) and perceived exhaustion (*ρ* = 0.375, *p* < 0.001) were weakly correlated with job demand, showing negative and positive correlations, respectively. Musculoskeletal pain, including lower back (*p*=0.003), upper back (*p*=0.004), shoulder (*p*=0.003), and neck pain (*p* < 0.001), significantly elevated job demand scores. Additionally, insufficient sleep during work (*p* < 0.001) and poor sleep quality at work (*p* < 0.001) were linked to higher demand. Experiences of bullying (*p* < 0.001), discrimination (*p* < 0.001), and aggressive patients (*p* < 0.001) also contributed to elevated job demand (Table 2).

The median job control score was 15 (IQR: 13–16). Self-perceived health (*ρ* = 0.269, *p* < 0.001) and perceived exhaustion (*ρ* = −0.355, *p* < 0.001) were weakly correlated with job control, showing positive and negative correlations, respectively. Students experiencing musculoskeletal pain, including lower back (*p*=0.046), upper back (*p*=0.011), shoulder (*p*=0.009), and neck pain (*p* < 0.001), reported diminished control. Insufficient sleep during work (*p*=0.023), poor sleep quality at work (*p* < 0.001), and poor sleep quality at rest (*p*=0.016) were linked to lower job control. Furthermore, experiences of bullying (*p*=0.035) and discrimination (*p*=0.016) negatively impacted job control (Table 2).

The median social support score was 16 (IQR: 14–18). Self-perceived health (*ρ* = 0.285, *p* < 0.001) and perceived exhaustion (*ρ* = −0.356, *p* < 0.001) were weakly correlated with social support, showing positive and negative correlations, respectively. Musculoskeletal pain, specifically upper back (*p*=0.020), shoulder (*p*=0.018), and neck region pain (*p* < 0.001), was linked to lower support scores. Insufficient sleep during work (*p* < 0.001), poor sleep quality at work (*p* < 0.001), and poor sleep quality at rest (*p*=0.006) further decreased perceived social support. Experiences of bullying (*p*=0.010) and discrimination (*p*=0.006) also significantly reduced social support levels (Table 2).

### 3.3. WAI

The overall WAI score was 39 (IQR: 36–42). Male students reported significantly higher WAI scores compared to female students (*p*=0.049). Self-perceived health (*ρ* = 0.531, *p* < 0.001) and perceived exhaustion (*ρ* = −0.407, *p* < 0.001) were moderately correlated with WAI scores, showing positive and negative correlations, respectively. Musculoskeletal pain, including lower back (*p*=0.005), upper back (*p* < 0.001), shoulder (*p* < 0.001), and neck pain (*p* < 0.001), was linked to decreased WAI. Additionally, insufficient sleep during work (*p* < 0.001) and poor sleep quality both at work (*p* < 0.001) and at rest (*p*=0.001) were associated with lower WAI scores. Experiences of bullying (*p*=0.001) and discrimination (*p*=0.003) also negatively impacted work ability (Table 2).

### 3.4. WAFCS

The participants exhibited higher levels of WFC compared to FWC. For WFC, the median score was 21 (IQR: 15–28), with notable concerns related to a lack of time for personal activities (Item 2, median = 5 [IQR: 4–7]) and irritability at home due to study obligations (Item 5, median = 5 [IQR: 3–6]). Additionally, challenges related to the negative impact of study obligations on family life (Items 3 and 4, median = 3 [IQR: 2–5]) were also evident. For FWC, the median score was 12 (IQR: 8–17), with participants reporting concerns related to being distracted by family worries or obligations while studying (Item 7, median = 3 [IQR: 2–5]) and the impact of personal and family commitments on study performance (Item 6, median = 3 [IQR: 2–5]) (Table 3).

WFC was weakly yet positively correlated with older age (*ρ* = 0.215, *p* < 0.001) and advanced study years (*ρ* = 0.178, *p* < 0.001). It was also significantly associated with full-time study mode (*p*=0.032), employment (*p*=0.006), and working in intensive care units (*p*=0.010). Self-perceived health (*ρ* = −0.302, *p* < 0.001) and perceived exhaustion (*ρ* = 0.436, *p* < 0.001) were moderately correlated with WFC, negatively and positively, respectively. Musculoskeletal pain (upper back, shoulder, and neck; all *p* ≤ 0.001), insufficient sleep and poor sleep quality at both work and rest (all *p* < 0.001), and experiences of bullying and discrimination at clinical practice (all *p* ≤ 0.001) were significantly associated higher WFC scores (Table 4).

FWC was weakly yet positively correlated with older age (*ρ* = 0.307, *p* < 0.001) and advanced study years (*ρ* = 0.118, *p*=0.005). It was also significantly associated with full-time study mode (*p* < 0.001), employment (*p* < 0.001), and full-time workload (*p*=0.021). Self-perceived health (*ρ* = −0.262, *p* < 0.001) and perceived exhaustion (*ρ* = 0.358, *p* < 0.001) were moderately correlated with FWC, negatively and positively, respectively. Musculoskeletal pain (lower back, upper back, and shoulder, neck; all *p* < 0.001), insufficient sleep and poor sleep quality at both work and rest (all *p* < 0.001), and experiences of bullying and discrimination in clinical practice (all *p* ≤ 0.001) were significantly associated with higher FWC scores (Table 4).

### 3.5. Nursing Training Feedback

Participants reported moderate satisfaction with their clinical training, with a median expectation alignment score of 11 (IQR: 7–15). There was moderate agreement that clinical practice met their expectations (median = 5 [IQR: 3–8]) and complemented what they had learned during their studies (median = 6 [IQR: 3–8]). In terms of ENT, there was a strong tendency to remain in healthcare after graduation (median = 9 [IQR: 6–10]) and to successfully complete their current study program (median = 9 [IQR: 7–10]), resulting in an overall endurance score of 27 (IQR: 21–30) (Table 5).

TEA was weakly and negatively correlated with study year (*ρ* = −0.146, *p* < 0.001) and perceived exhaustion (*ρ* = −0.298, *p* < 0.001), while positively correlated with self-perceived health (*ρ* = 0.254, *p* < 0.001). Musculoskeletal pain in the lower back (*p*=0.029), upper back (*p*=0.022), shoulder (*p*=0.009), and neck (*p*=0.013) were significantly associated with lower TEA scores. Similarly, insufficient sleep and poor sleep quality at both work and rest (all *p* < 0.001), along with experiences of bullying (*p* < 0.001) and discrimination (*p*=0.020), were significantly associated with lower TEA scores (Table 6).

ENT was significantly higher among employed students (*p*=0.001) and positively correlated with self-perceived health (*ρ* = 0.274, *p* < 0.001), while negatively correlated with perceived exhaustion (*ρ* = −0.358, *p* < 0.001). Musculoskeletal pain in the upper back (*p*=0.003), shoulder (*p*=0.002), and neck (*p*=0.015) was associated with lower ENT. Likewise, sleep insufficiency and poor sleep quality at both work and rest (all *p* < 0.001), as well as experiences of bullying (*p* < 0.001) and discrimination (*p*=0.032), were significantly associated with lower ENT scores (Table 6).

### 3.6. Professional Intentions

The majority of participants (87.9%) expressed an intention to increase their qualifications in nursing, while 61.6% showed interest in pursuing qualifications outside of nursing. Regarding professional exodus, 55.6% reported having considered quitting or giving up the nursing profession at least once, and 48.9% had considered pursuing a different qualification or profession (Table 5).

Increasing qualifications in nursing was significantly associated part-time study mode (*p*=0.003) and weakly correlated with study year (*ρ* = −0.133, *p*=0.002). Being exposed to death (89.3% vs. 76.4%; *p*=0.005), human suffering (88.5% vs. 60%; *p*=0.023), problematic patients (88.2% vs. 33.3%; *p*=0.040), and discrimination (93.3% vs. 86.4%; *p*=0.042) were significantly associated with intentions to increase qualifications in nursing (Table 7).

Increasing qualifications outside of nursing was weakly correlated with self-perceived health (*ρ* = −0.140, *p*=0.001) and perceived exhaustion (*ρ* = 0.188, *p* < 0.001), negatively and positively, respectively. Being exposed to discrimination (71.9% vs. 58.5%; *p*=0.008) were significantly associated with intentions to increase qualifications outside of nursing (Table 7).

Quitting the nursing profession was negatively and weakly correlated with self-perceived health (*ρ* = −0.239, *p* < 0.001) and positively and moderately correlated with perceived exhaustion (*ρ* = 0.444, *p* < 0.001). Musculoskeletal pain at lower back (*p*=0.041), upper back (*p*=0.004), shoulder (*p*=0.023), and neck region (*p* < 0.001) were associated with intentions to quit the nursing profession. Likewise insufficient sleep at work (*p* < 0.001) and rest (*p*=0.003) and poor sleep at both (*p* < 0.001 and *p*=0.014, respectively) and experiencing bullying (*p* < 0.001) and aggressive patients (*p*=0.036) were significantly associated with intentions of professional exodus (Table 7).

Intentions to start a different qualification or profession were significantly associated with full-time study mode (*p*=0.030) and being unemployed (*p*=0.004). They were weakly and negatively correlated with self-perceived health (*ρ* = −0.225, *p* < 0.001) and moderately and positively correlated with perceived exhaustion (*ρ* = 0.352, *p* < 0.001). Insufficient sleep during work (*p*=0.031), poor sleep quality at work (*p* < 0.001), and experiences of bullying (*p* < 0.001) were also significantly linked to intentions of pursuing another profession (Table 7).

### 3.7. Correlation of JDCS, WAI, WAFCS, and Training Feedback and Professional Intentions

Nonparametric (Spearman's *ρ*) correlation analyses were performed between occupational psychosocial determinants (JDCS, WFC, FWC, and WAI) and training feedback (TEA and ENT) as well as professional intentions. TEA and ENT were weakly and negatively correlated with job demand (*ρ* = −0.157 and −0.192, respectively) and WFC indicators, including WFC (*ρ* = −0.330 and −0.214), FWC (*ρ* = −0.254 for both), and WAFCS (*ρ* = −0.327 and −0.255). In contrast, both TEA and ENT were weakly and positively correlated with job control (*ρ* = 0.207 and 0.208) and moderately with social support (*ρ* = 0.634 and 0.325). Additionally, moderate positive correlations were observed with WAI (*ρ* = 0.309 and 0.378) (Table 8).

Regarding professional intentions, quitting the nursing profession and starting a different profession were weakly and positively correlated with job demand (*ρ* = 0.215 and 0.141), WFC (*ρ* = 0.326 and 0.270), FWC (*ρ* = 0.269 and 0.191), and WAFCS (*ρ* = 0.332 and 0.255). Contrarily, both were weakly to moderately negatively correlated with job control (*ρ* = −0.231 and −0.182), social support (*ρ* = −0.357 and −0.355), and WAI (*ρ* = −0.339 and −0.329). Increasing non-nursing qualifications showed similar weak positive correlations with job demand, WFC, FWC, and WAFCS and weak negative correlations with WAI. In contrast, increasing nursing qualifications had negligible correlations with most determinants, except for very weak positive correlations with job demand (*ρ* = 0.087) and WAI (*ρ* = 0.110) (Table 8).

### 3.8. Linear Regression of Training Feedback Predictors

Multilinear regression models were established to identify the key predictors of TEA and ENT among Czech nursing students. All models were adjusted for gender, age, BMI, secondary school type, study program, study year, study form, workload, and workplace.

For TEA, social support emerged as the strongest positive predictor (*β* = 1.06, 95% CI: 0.88–1.23, *R*^2^ = 40.8%), followed by WAI (*β* = 0.21, 95% CI: 0.09–0.34, *R*^2^ = 10.7%) and self-perceived health (*β* = 0.21, 95% CI: 0.10–0.41, *R*^2^ = 8.3%). Contrarily, perceived exhaustion (*β* = −0.36, 95% CI: −0.53 to −0.18, *R*^2^ = 12.3%), WFC (*β* = −0.21, 95% CI: −0.29 to −0.13, *R*^2^ = 15.6%), and FWC (*β* = −0.19, 95% CI: −0.28 to −0.10, *R*^2^ = 13%) were key negative predictors of TEA (Table 9).

For ENT, WAI was the strongest positive predictor (*β* = 0.47, 95% CI: 0.36–0.59, *R*^2^ = 23.2%), followed by self-perceived health (*β* = 0.46, 95% CI: 0.27–0.66, *R*^2^ = 10.8%) and social support (*β* = 0.46, 95% CI: 0.25–0.67, *R*^2^ = 9.6%). In contrast, perceived exhaustion (*β* = −0.58, 95% CI: −0.75 to −0.41, *R*^2^ = 18%), FWC (*β* = −0.27, 95% CI: −0.36 to −0.19, *R*^2^ = 15.9%), WFC (*β* = −0.16, 95% CI: −0.24 to −0.07, *R*^2^ = 7.9%), and job demand (*β* = −0.51, 95% CI: −0.81 to −0.21, *R*^2^ = 7.1%) were key negative predictors of ENT (Table 9).

### 3.9. Logistic Regression of Professional Intentions Predictors

Multivariable logistic regression models were established for professional intentions. They were also adjusted for sociodemographic, education- and employment-related attributes.

For increasing nursing qualifications, social support was the only significant positive predictor (AOR = 1.22, 95% CI: 1.04–1.41, *R*^2^ = 15.9%). In contrast, for increasing non-nursing qualifications, perceived exhaustion (AOR = 1.13, 95% CI: 1.04–1.23, *R*^2^ = 9.9%) and lower WAI (AOR = 0.93, 95% CI: 0.88–0.99, *R*^2^ = 8.4%) were significant predictors (Table 10).

Regarding quitting the nursing profession, the promoters included higher perceived exhaustion (AOR = 1.23, 95% CI: 1.13–1.34, *R*^2^ = 15.3%), increased job demand (AOR = 1.17, 95% CI: 1.04–1.33, *R*^2^ = 5.2%), greater WFC (AOR = 1.08, 95% CI: 1.04–1.12, *R*^2^ = 10.9%), and greater FWC (AOR = 1.07, 95% CI: 1.03–1.11, *R*^2^ = 8.3%). On the other side, the barriers included lower WAI (AOR = 0.87, 95% CI: 0.82–0.92, *R*^2^ = 14.7%), poor self-perceived health (AOR = 0.88, 95% CI: 0.81–0.96, *R*^2^ = 6.8%), and lower social support (AOR = 0.84, 95% CI: 0.77–0.92, *R*^2^ = 9.3%) (Table 10).

Intentions to start a different study or profession were promoted by higher perceived exhaustion (AOR = 1.19, 95% CI: 1.10–1.28, *R*^2^ = 13.4%), greater WFC (AOR = 1.07, 95% CI: 1.03–1.11, *R*^2^ = 10%), and greater FWC (AOR = 1.08, 95% CI: 1.04–1.12, *R*^2^ = 10.9%). Contrarily, the barriers to pursuing a different academic or professional path were higher WAI (AOR = 0.86, 95% CI: 0.80–0.91, *R*^2^ = 18.2%), better self-perceived health (AOR = 0.86, 95% CI: 0.80–0.94, *R*^2^ = 9.6%), and stronger social support (AOR = 0.87, 95% CI: 0.79–0.95, *R*^2^ = 8.4%) (Table 10).

## 4. Discussion

This national survey of Czech nursing students explores structural, psychosocial, and health-related factors that may shape their training experience and professional aspirations. The study considers WFC, job demands, job control, and social support, acknowledging that early-career experiences and perceived support could play a role in workforce retention. Nursing student dropout represents a substantial loss of human and financial capital, with late-stage dropout being especially problematic due to extensive investments without outcomes [25]. Replacement costs for healthcare organizations are estimated to be three times the average salary of nurses, alongside uncounted impacts on staff and work disruption [3]. Given this, it is essential not only to expand our focus beyond simply training more staff but also to include the retention strategies for recently graduated professionals. Ensuring long-term sustainability requires a balanced approach that emphasizes training, retention, and systemic reform [26]. Furthermore, fostering evidence-based improvements in nursing education through adaptive learning environments can enhance academic outcomes and build a resilient and sustainable nursing workforce.

### 4.1. Nursing Training Feedback

The alignment of training expectations among nursing students in our study was found to be moderate, reflected by a composite median score of 11 (IQR = 7–15). This suggests that while students generally perceived their clinical practice experiences as meeting their expectations, there is clear potential for improvement in bridging the gap between anticipated and actual experiences. Lin et al. [27] identified that one of the primary motivations for nursing students choosing the profession was the strong job prospects, particularly the perceived ease of securing employment. However, unmet expectations, a disconnect between theoretical learning and clinical practice, and feelings of career misalignment contribute significantly to early dropout rates [25].

### 4.2. Role of Social Support

Our findings underscore the importance of social support in shaping nursing students' training experiences. Specifically, higher perceived social support was associated with improved alignment between training expectations and actual experiences. This relationship reinforces previous research demonstrating that inadequate support from faculty, negative clinical placements, and weak peer or mentor networks can undermine students' motivation and contribute to attrition from nursing education or the profession itself [25, 28]. Strengthening supportive structures in both academic and clinical settings may therefore be a key strategy in enhancing student retention and satisfaction. Empowerment also emerged as a significant determinant of professional engagement, with research indicating that empowered nurses demonstrate improved patient care, reduced burnout, and higher job satisfaction. Additionally, empowered professionals are more likely to facilitate patient-centered care and adopt effective work strategies, reinforcing the broader benefits of empowerment for both individuals and the nursing profession [2]. To enhance nursing students' training experiences and professional resilience, targeted strategies such as peer mentoring, structured faculty advising, and the reinforcement of institutional support networks are recommended. Regular assessment of these support mechanisms is necessary to ensure their continued relevance and effectiveness in addressing the evolving challenges faced by nursing students. Strengthening these initiatives may contribute to a more sustainable and engaged nursing workforce.

### 4.3. Determinants of Endurance in Nursing Education

Our findings show that endurance in nursing education was notably high among participants, with a composite median score of 27 (IQR = 21–30). This suggests that many nursing students have a strong capacity to manage the academic, physical, and emotional pressures of their training. The low levels of reported dropout intentions (median = 10; IQR = 8–10) further reflect their professional commitment and determination to continue in the field. These findings support Wongkoey's [29] view of endurance as the ability to cope with stress, fatigue, and personal challenges while staying focused on the goal of becoming a nurse.

This level of endurance is reassuring, especially in light of the well-documented challenges nursing students face during clinical placements—such as emotional strain, high workloads, and disrupted work–life balance. While our results highlight students' resilience, they also suggest that the right support structures are the key. Literature points to the importance of supportive learning environments, strong peer and faculty networks, and inclusive academic cultures in helping students stay motivated and committed during difficult periods [2, 30].

To maintain and build on this endurance, targeted strategies are needed. These might include structured mentorship, opportunities for leadership development, and more flexible learning or working arrangements to better accommodate students' lives outside of education [26]. Alongside this, fostering a sense of empowerment through participatory management and inclusive decision-making can make a real difference. Tackling issues like WFC, poor clinical supervision, and challenging working conditions is crucial—not only to support students throughout their training but also to improve long-term retention and engagement in the nursing profession.

### 4.4. The Influence of Exhaustion

Despite the overall high levels of endurance, our analysis identified exhaustion as a significant negative predictor (AOR = −0.247; 95% CI: −0.470–-0.025; *p*=0.030), suggesting that prolonged fatigue undermines students' capacity to sustain engagement in their education. Exhaustion is well-documented in the literature as a precursor to burnout, which is frequently associated with high job demands, stressful working conditions, excessive workloads, WFCs, and poor interpersonal relationships with colleagues [16]. Burnout has broader implications beyond individual endurance; it is linked to diminished professional engagement, increased turnover intentions, and a heightened risk of errors in patient care [3, 31]. Importantly, burnout is not only experienced by students but also by their clinical mentors, whose well-being directly impacts the quality of nursing education and patient safety. Evidence suggests that fostering positive work environments, involving nurses in decision-making, and promoting supportive leadership can mitigate emotional and physical exhaustion, thereby improving both endurance and workforce retention [31].

### 4.5. WFC and Its Impact

FWC emerged as another significant factor negatively influencing endurance in nursing education (AOR = −0.133; 95% CI: −0.246–−0.020; *p*=0.021). Students who reported higher levels of FWC conflict struggled to balance academic and personal responsibilities, which in turn reduced their ability to sustain long-term commitment to the profession. This finding is consistent with prior research, which indicates that nurses with low FWC demonstrate greater work investment, while those experiencing such conflicts are more likely to deliver poorer patient care and face increased turnover risk [32]. Addressing these challenges requires institutional policies that support work–life balance, including flexible scheduling and structured mentorship.

### 4.6. Work Ability and Its Role in Endurance

In our study, the WAI showed a strong positive relationship with endurance (AOR = 0.309; 95% CI: 0.157–0.462; *p* < 0.001), emphasizing the importance of maintaining both physical and mental health to sustain nursing students' engagement. This is consistent with the findings of Martinez et al. [33], who demonstrated that impaired work ability is closely linked to the intention to leave the profession. Factors such as job strain, effort-reward imbalance, musculoskeletal risks, and insomnia were significantly associated with nurses' intention to leave the profession. Furthermore, overall health was reported to be negatively impacted by low job control, high psychological demands, frequent night shifts, and a lack of workplace justice [32].

### 4.7. Factors Influencing Nursing Students' Career Intentions

Our findings indicate that a substantial proportion of nursing students express a strong commitment to advancing their professional development. The majority (87.9%, *n* = 466) expressed interest in pursuing advanced nursing qualifications (median = 3; IQR = 2–4), demonstrating a strong motivation to develop their professional skills and advance within the field. However, it is noteworthy to mentioned that a significant portion of the study sample expressed intentions to explore non-nursing qualifications (61.6%, *n* = 327; median = 2; IQR = 1–3), leave the nursing profession entirely (55.6%, *n* = 304; median = 2; IQR = 1–3), or transition to other professions (48.9%, *n* = 268; median = 1; IQR = 1–2). These findings align, in part, with research by Lin et al. [27], which identified that a significant number of nursing students (50.6%) had familial ties to the profession and were motivated by positive perceptions of nursing (91.8%) and job security (46.9%). Despite this, a considerable proportion of Lin et al.'s participants expressed dissatisfaction or uncertainty about their career path, with 56.3% considering switching to other fields, 60% favoring nonhealth careers, and 45.2% exploring additional health-related training.

Our analysis highlights the critical role of social support in shaping students' career intentions. Increased social support was associated with a 30.8% higher likelihood of students pursuing further nursing qualifications, underscoring the importance of a supportive learning and clinical environment in fostering long-term professional commitment. Interestingly, students experiencing WFC were also more inclined to seek advanced nursing qualifications. These findings align with previous studies, which emphasize the importance of supportive learning environments in influencing students' career intentions. For example, Zhang et al. [34] found a correlation between supportive learning environments, such as supervision and peer interaction, and nursing students' intentions to pursue a future in the profession. Furthermore, motivation and academic achievement play key roles in encouraging students to reach higher academic goals. This may suggest that, for some students, career progression is perceived as a means to improve work–life balance and enhance professional stability.

However, not all students in our sample expressed intentions to remain in nursing. Specifically, the intention to pursue non-nursing qualifications was significantly influenced by perceived exhaustion, with a 14.5% increase in the likelihood of students considering non-nursing fields as they experienced higher levels of fatigue and dissatisfaction with their nursing roles. This is in line with evidence indicating that work experience and clinical placements play a crucial role in shaping career confidence. Previous studies suggest that students with greater exposure to positive clinical experiences are more likely to feel competent and remain motivated to complete their training [35]. Furthermore, students demonstrating higher levels of resilience, teamwork, and academic motivation tend to aspire toward greater academic achievement and professional growth [29].

Regarding students' intentions to leave the nursing profession, our model accounted for 22.9% of the variability. Perceived exhaustion and declining work ability emerged as significant predictors, with students experiencing higher levels of exhaustion being 13.9% more likely to consider leaving the profession. Additionally, a decrease in work ability was associated with a 9.2% increase in the likelihood of exiting nursing, while students with lower work ability were also 10.9% more likely to transition to a different career path. These findings are consistent with Canzan et al. [1] who identified several key factors contributing to nursing student attrition, including perceived unfitness for the profession, insufficient institutional support, and a disconnect between initial career expectations and the realities of nursing practice. Dissatisfaction with clinical placements and inadequate mentorship were also highlighted as influential factors. Alghtany et al. [28] identified that one in six nursing students expresses the desire to leave the profession within their first 2 years of employment, often due to similar range of challenges. Collectively, these results underscore the need for targeted interventions aimed at enhancing student support mechanisms, mitigating exhaustion, and promoting sustainable career pathways to strengthen workforce retention in the nursing profession.

## 5. Conclusion

This study revealed a complex interplay of psychosocial, structural, and health-related factors influencing nursing students' career intentions in the Czech Republic. Social support emerged as a significant protective factor, fostering alignment between academic training and clinical realities and promoting perseverance throughout nursing education. Students with higher perceived social support were more likely to express intentions to remain in the profession and pursue advanced qualifications.

In contrast, WFC and emotional exhaustion were strongly associated with reduced endurance in training and lower work ability. These stressors increased the likelihood of students considering alternative career pathways, including leaving the nursing profession entirely or pursuing non-nursing qualifications. Health-related issues—particularly musculoskeletal pain and sleep disturbances—further contributed to negative career outlooks.

Negative experiences during clinical placements, such as bullying and discrimination, were also associated with professional disengagement, highlighting the influence of workplace culture on early-career decision-making. Additionally, high job demands were significantly related to intentions to leave the profession, reinforcing the need to monitor workload and stress levels during clinical education.

Taken together, these findings point to several practical implications. Strengthening institutional and peer support, addressing workplace stressors, and enhancing the quality of clinical placements are critical for improving student well-being and fostering long-term professional commitment. Promoting a positive and inclusive clinical learning environment, supported by active mentorship and clear channels for addressing concerns, may help increase retention and build a more resilient nursing workforce.

### 5.1. Importance and Relevance

The study findings highlight the urgent need for institutional and systemic interventions to enhance nursing education and workforce retention. Social support mechanisms, including structured mentorship and peer networks, are crucial to bridge the gap between academic training and clinical practice. Addressing stressors like exhaustion and WFC through flexible schedules, counseling, and workload adjustments can help improve resilience and well-being among nursing students. Policymakers should prioritize investments in evidence-based reforms that create supportive and empowering educational settings. Additionally, addressing systemic challenges such as workload pressures and burnout in clinical environments can positively influence retention rates, benefiting both students and the broader healthcare workforce.

### 5.2. Limitation

While this study provides valuable insights, certain limitations should be acknowledged. First, the use of self-reported data may introduce bias, as participants could overestimate or underestimate their job demands, support levels, or career intentions. Second, the cross-sectional nature of the study limits the ability to establish causal relationships between variables, as data were collected at a single point in time. Third, the sample was restricted to nursing students from the Czech Republic, which may limit the generalizability of findings to other countries or cultural settings. Future research should incorporate mixed methods and longitudinal designs with more diverse samples to examine these determinants over time and across various educational levels and universities and national study on the Czech nursing workforce, including both active nurses and those who have left the profession to understand the underlying the complexity behind the premature departure of nursing workforce.

## Figures and Tables

**Table 1:** Sociodemographic and anamnestic characteristics of Czech nursing students participating in the SYRI survey, August–November 2023 (*N* = 559).

	Variable	Outcome	Frequency (%)
Sociodemographic	Gender	Female	514 (91.9%)
		Male	40 (7.2%)
		Diverse	5 (0.9%)
	Age group	≤ 21 years old	326 (58.4%)
		> 21 years old	232 (41.6%)
	BMI level	Underweight	34 (6.1%)
		Normal weight	312 (56.2%)
		Overweight	134 (24.1%)
		Obesity	75 (13.5%)

Education	Secondary school	General secondary school	99 (17.7%)
		Specialized secondary school (nonhealth)	59 (10.6%)
		Specialized secondary school (healthcare)	401 (71.7%)
	Highest obtained degree	Secondary school	498 (89.1%)
		Healthcare college (VOŠ)	24 (4.3%)
		University degree program	37 (6.6%)
	Study program	Bachelor's degree program (Bc.)	544 (97.3%)
		Master's degree program (Mgr.)	15 (2.7%)
	Study year	First year	146 (26.1%)
		Second year	191 (34.2%)
		Third year	215 (38.5%)
		Fourth year	7 (1.3%)
	Study form	Full time	402 (71.9%)
		Part time	157 (28.1%)

Employment	Employment status	Employed^ꝉ^	261 (53.3%)
		Unemployed	298 (46.7%)
	^ꝉ^Workload	Part time (≤ 20 h per week)	110 (42.1%)
		Part time (> 20 h per week)	57 (21.8%)
		Full time (40 h per week)	94 (36%)
	^ꝉ^Workplace	Intensive care unit (ICU)	57 (21.8%)
		Standard care department	163 (62.5%)
		Other	41 (15.7%)

Prc	Self-perceived health	Median and IQR	14 (11–16)
	Perceived exhaustion	Median and IQR	10 (8–13)

Musculoskeletal pain	Lower back pain	Yes	444 (81.3%)
		No	102 (18.7%)
	Upper back pain	Yes	238 (45%)
		No	291 (55%)
	Shoulder region pain	Yes	277 (52.1%)
		No	255 (47.9%)
	Neck region pain	Yes	372 (68.8%)
		No	169 (31.2%)

Sleep quality	Sufficient sleep during work	Enough	389 (69.6%)
		Not enough	170 (30.4%)
	Sufficient sleep during rest	Enough	164 (29.3%)
		Not enough	395 (70.7%)
	Sleep quality during work^∗^	High quality	117 (20.9%)
		Low quality	243 (43.5%)
	Sleep quality during rest^∗^	High quality	341 (61%)
		Low quality	81 (14.5%)

Challenging experiences	Death	Yes	496 (88.7%)
		No	63 (11.3%)
	Human suffering	Yes	548 (98%)
		No	11 (2%)
	Aggressive patients	Yes	525 (93.9%)
		No	34 (6.1%)
	Problematic patients	Yes	555 (99.3%)
		No	4 (0.7%)
	Bullying	Yes	236 (42.2%)
		No	323 (57.8%)
	Discrimination	Yes	128 (22.9%)
		No	431 (77.1%)

^∗^Missed values are those who reported “average” sleep quality.

^ꝉ^The employed subgroup.

**Table 2:** Job demand, control, social support, and work ability of Czech nursing students participating in the SYRI survey, stratified by sociodemographic and anamnestic characteristics, August–November 2023 (N = 559).

	Variable	Outcome	Demand: median (IQR)	*Sig.*	Control: median (IQR)	*Sig.*	Support: median (IQR)	*Sig.*	Work ability: median (IQR)	*Sig.*
Sociodemographic	Gender	Female	12 (10–13)	0.307	15 (13–16)	0.196	16 (13–18)	0.298	39 (36–42)	**0.049**
		Male	12 (10–14)		16 (14–17)		16 (15–18)		41 (38–44)	
		Diverse	14 (10.5–14.5)		15 (14.5–17)		17 (15–20)		40 (37–41)	
	Age group	≤ 21 years old	12 (11–13)	0.140	15 (13–17)	0.143	16 (14–18)	0.108	39 (36–42)	0.586
		> 21 years old	12 (10–13)		15 (13–16)		15 (13–18)		40 (36–42)	
		Correlation (*Spearman's rho*)	−0.084	**0.047**	−0.039	0.358	−0.068	0.111	0.027	0.528
	BMI level	Underweight	12 (11–14)	0.141	15 (13–15.25)	0.765	17 (14–18)	0.483	39 (35–40.75)	0.266
		Normal weight	12 (10–13)		15 (13–17)		16 (14–18)		40 (37–43)	
		Overweight	12 (10–13)		15 (13.75–16)		16 (13.75–18)		39 (36–42)	
		Obesity	12 (11–14)		15 (13–17)		15 (13–17)		40 (35.5–41)	
		Correlation (*Spearman's rho*)	0.008	0.854	0.039	0.356	−0.027	0.521	−0.008	0.859

Education	Secondary school	General secondary school	11 (10–12)	**<** **0.001**	15 (13–17)	0.628	16 (14–18)	0.148	40 (37–43)	0.211
		Specialized secondary school (nonhealth)	10 (9–13)		15 (14–16)		17 (14–18)		40 (35–42)	
		Specialized secondary school (healthcare)	12 (11–13)		15 (13–16)		16 (13–18)		39 (36–42)	
	Highest obtained degree	Secondary school	12 (10–13)	0.155	15 (13–17)	0.577	16 (14–18)	0.114	39 (36–42)	0.394
		Healthcare college (VOŠ)	11 (9–12.75)		14 (13–16)		13.5 (12–18)		38.5 (33–42.25)	
		University degree program	12 (10–13)		15 (13–16)		17 (14.5–18.5)		41 (36–44)	
		Correlation (*Spearman's rho*)	−0.079	0.062	−0.021	0.628	0.016	0.705	0.013	0.762
	Study program	Bachelor's degree program (Bc.)	12 (10–13)	0.539	15 (13–16)	0.747	16 (13–18)	**0.043**	39 (36–42)	0.350
		Master's degree program (Mgr.)	12 (11–13)		15 (13–16)		17 (15–20)		41 (36–44.5)	
	Study year	First year	12 (10–13.25)	0.326	15 (13–17)	0.706	17 (15–19)	**<** **0.001**	39 (36–43)	0.469
		Second year	12 (11–13)		15 (13–17)		16 (14–18)		39 (36–42)	
		Third year	12 (10–13)		15 (13–16)		15 (12–17)		40 (36–42)	
		Fourth year	12 (9–14)		14 (14–18)		17 (12–18)		41 (40–43)	
		Correlation (*Spearman's rho*)	−0.064	0.129	−0.037	0.388	−0.274	**<** **0.001**	−0.015	0.718
	Study form	Full time	12 (11–13)	0.065	15 (13–17)	0.352	16 (13–17)	**0.005**	39 (36–42)	0.196
		Part–time	11 (10–13)		15 (14–16)		17 (14–18)		40 (37–43)	

Employment	Employment status	Employed^ꝉ^	12 (10–13)	0.148	15 (14–17)	0.073	16 (13–18)	0.813	40 (36–42)	0.332
		Unemployed	12 (11–13)		15 (13–16)		16 (14–18)		39 (36–42)	
	^ꝉ^Workload	Part time (≤ 20 h per week)	12 (10–13)	0.946	15 (14–16)	0.503	15 (13–17)	**0.022**	40 (36.25–41)	0.715
		Part time (> 20 h per week)	12 (10–13)		15 (14–17)		15 (12.5–17)		39 (37–44)	
		Full time (40 h per week)	11.5 (10–14)		15 (14–17)		17 (14–18)		40 (36–42)	
	^ꝉ^Workplace	Intensive care unit (ICU)	12 (10–13)	0.904	15 (13–16)	0.954	16 (13–18)	0.307	40 (37–43)	0.462
		Standard care department	12 (10–14)		16 (14–17)		16 (15–18)		39 (36–42)	
		Other	14 (10.5–14.5)		15 (14.5–17)		17 (15–20)		41 (37–43)	

Prc	Self-perceived health	Correlation (*Spearman's rho*)	−0.252	**<** **0.001**	0.269	**<** **0.001**	0.288	**<** **0.001**	0.531	**<** **0.001**
	Perceived exhaustion	Correlation (*Spearman's rho*)	0.375	**<** **0.001**	−0.355	**<** **0.001**	−0.356	**<** **0.001**	−0.542	**<** **0.001**

Musculoskeletal pain	Lower back Pain	Yes	12 (11–13)	**0.003**	15 (13–16)	**0.046**	16 (13–18)	0.088	39 (36–42)	**0.005**
		No	11 (10–12)		16 (13.25–17)		16 (14.25–18)		41 (37–43)	
	Upper back Pain	Yes	12 (11–13.75)	**0.004**	15 (13–16)	**0.011**	15 (13–17)	**0.020**	38 (34–41)	**<** **0.001**
		No	12 (10–13)		15 (13.5–17)		16 (14–18)		40 (37–43)	
	Shoulder region pain	Yes	12 (11–14)	**0.003**	15 (13–16)	**0.009**	15 (13–17)	**<** **0.001**	38 (35–41)	**<** **0.001**
		No	11 (10–13)		15 (13–17)		16 (14–18)		41 (37–43)	
	Neck region pain	Yes	12 (11–13)	**<** **0.001**	14.5 (13–16)	**<** **0.001**	15 (13–17.25)	**<** **0.001**	38.5 (35–41)	**<** **0.001**
		No	11 (10–13)		16 (14–17)		16 (15–18)		41 (39–44)	

Sleep quality	Enough sleep/work	Enough	11 (10–12.25)	**<** **0.001**	16 (14–17)	**<** **0.001**	17 (14.75–18)	**<** **0.001**	41 (38–43)	**<** **0.001**
		Not enough	12 (11–14)		14 (13–16)		16 (13–17)		39 (35–42)	
	Enough sleep/rest	Enough	12 (10–13)	0.270	15 (14–17)	**0.023**	16 (14–18)	**0.005**	40 (37–43)	**<** **0.001**
		Not enough	12 (11–13)		14.5 (13–16)		15 (13–17.75)		38 (33.25–41)	
	Sleep quality/Work	High quality	11 (10–12)	**<** **0.001**	16 (15–17)	**<** **0.001**	17 (15–19)	**<** **0.001**	41 (39–44)	**<** **0.001**
		Low quality	12 (11–14)		14 (13–15)		15 (12–17)		38 (33–41)	
	Sleep quality/rest	High quality	12 (10–13)	0.139	15 (14–17)	**0.004**	16 (14–18)	**0.002**	40 (37–43)	**<** **0.001**
		Low quality	12 (11–14)		14 (13–16)		15 (13–17)		37 (30.5–40)	

Challenging experiences	Death	Yes	12 (10.25–13)	0.168	15 (13–16)	0.265	16 (14–18)	0.463	40 (36–42)	0.277
		No	11 (10–13)		15 (14–17)		16 (13–17)		39 (34–42)	
	Human suffering	Yes	12 (10–13)	0.500	15 (13–16)	0.611	16 (14–18)	0.138	39.5 (36–42)	0.069
		No	12 (11–13)		16 (13–17)		14 (10–17)		37 (30–41)	
	Aggressive patients	Yes	12 (11–13)	**<** **0.001**	15 (13–16)	0.572	16 (14–18)	0.800	39 (36–42)	0.308
		No	10 (9–11)		15 (13–17)		16.5 (12.75–18.25)		41 (36–43.25)	
	Problematic patients	Yes	12 (10–13)	0.503	15 (13–16)	0.716	16 (14–18)	0.503	39 (36–42)	**0.039**
		No	11 (10.25–12.5)		15.5 (13–18)		17.5 (12.5–18.75)		30.5 (26.25–38.5)	
	Bullying	Yes	12 (11–14)	**<** **0.001**	14 (13–16)	**<** **0.001**	15 (12–17)	**<** **0.001**	38 (34–41)	**<** **0.001**
		No	11 (10–13)		15 (14–17)		17 (15–18)		40 (37–43)	
	Discrimination	Yes	13 (11–14)	**<** **0.001**	14.5 (13–16)	**0.035**	15 (13–17)	**0.010**	38 (34–41)	**<** **0.001**
		No	12 (10–13)		15 (14–16)		16 (14–18)		40 (37–43)	

*Note:* Mann–Whitney test (*U*), Kruskal–Wallis test (*H*), and nonparametric correlation (Spearman's *rho*) were conducted with a significance level (*Sig*.) < 0.05. Bold values represent statistically significant values (values below *p* 0.05).

^ꝉ^The employed subgroup.

**Table 3:** Work and family conflict scale (WAFCS) of Czech nursing students participating in the SYRI survey, August–November 2023 (*N* = 559).

#	Item	Very strongly disagree	Strongly disagree	Disagree	Neither agree nor disagree	Agree	Strongly agree	Very strongly agree	Median (IQR)
1	My clinical practice prevents me from spending quality time with my family	38 (6.8%)	80 (14.3%)	73 (13.1%)	97 (17.4%)	110 (19.7%)	54 (9.7%)	107 (19.1%)	4 (3–6)
2	At the end of the day, I have no time to do things I would like to do (such as housework and leisure activities)	25 (4.5%)	53 (9.5%)	39 (7%)	59 (10.6%)	107 (19.1%)	104 (18.6%)	172 (30.8%)	5 (4–7)
3	My family suffers because of my study obligations	113 (20.2%)	93 (16.6%)	86 (15.4%)	78 (14%)	67 (12%)	50 (8.9%)	72 (12.9%)	3 (2–5)
4	My study obligations have a negative impact on my family life	99 (17.7%)	105 (18.8%)	78 (14%)	75 (13.4%)	67 (12%)	61 (10.9%)	74 (13.2%)	3 (2–5)
5	Due to study obligations, I am often irritable at home	49 (8.8%)	66 (11.8%)	79 (14.1%)	65 (11.6%)	75 (13.4%)	94 (16.8%)	131 (23.4%)	5 (3–6)
Work-to-Family Conflict (WFC)		Item 1 + Item 2 + Item 3 + Item 4 + Item 5							21 (15–28)
6	My personal and family commitments limit my study performance	93 (16.6%)	101 (18.1%)	87 (15.6%)	76 (13.6%)	71 (12.7%)	68 (12.2%)	63 (11.3%)	3 (2–5)
7	I am often distracted by family worries or obligations while studying	91 (16.3%)	110 (19.7%)	106 (19%)	73 (13.1%)	66 (11.8%)	60 (10.7%)	53 (9.5%)	3 (2–5)
8	I would be a better student if I did not have a family	345 (61.7%)	56 (10%)	37 (6.6%)	30 (5.4%)	28 (5%)	20 (3.6%)	43 (7.7%)	1 (1–3)
9	My family has a negative impact on my daily study obligations	358 (64%)	96 (17.2%)	37 (6.6%)	26 (4.7%)	15 (2.7%)	13 (2.3%)	14 (2.5%)	1 (1–2)
10	It is difficult to concentrate on practice because family obligations exhaust me	291 (52.1%)	114 (20.4%)	49 (8.8%)	52 (9.3%)	21 (3.8%)	10 (1.8%)	22 (3.9%)	1 (1–3)
Family-to-work conflict (FWC)		Item 6 + Item 7 + Item 8 + Item 9 + Item 10							12 (8–17)

*Note:* Bold values represent statistically significant values (values below *p* 0.05).

**Table 4:** Work and family conflict scale (WAFCS) of Czech nursing students participating in the SYRI survey, stratified by sociodemographic and anamnestic characteristics, August–November 2023 (*N* = 559).

	Variable	Outcome	WFC: median (IQR)	*Sig.*	FWC: median (IQR)	*Sig.*	WAFCS: median (IQR)	*Sig.*
Sociodemographic	Gender	Female	21 (15–28)	0.750	12 (8–17)	0.193	34 (24.25–44)	0.445
		Male	20.5 (14.75–27.25)		11 (7.75–14.25)		33.5 (25.25–39)	
		Diverse	25 (24–25)		16 (13–17)		40 (38–42)	
	Age group	≤ 21 years old	20 (14–26)	**<** **0.001**	11 (7–15)	**<** **0.001**	31 (22–41)	**<** **0.001**
		> 21 years old	24 (17–29)		14 (10–20)		37 (28.75–47.25)	
		Correlation (*Spearman's rho*)	0.215	**<** **0.001**	0.307	**<** **0.001**	0.284	**<** **0.001**
	BMI level	Underweight	20 (13.5–27)	0.767	11.5 (9–18)	0.137	33.5 (24.5–44.25)	0.541
		Normal weight	21 (15–28)		11 (7–17)		33 (24–43)	
		Overweight	21 (15.25–27)		12 (8–17)		34 (23.5–43)	
		Obesity	21 (16.5–30)		13 (10–18.5)		35 (27–43.5)	
		Correlation (*Spearman's rho*)	0.052	0.217	0.039	0.357	0.046	0.279

Education	Secondary school	General secondary school	22 (14.5–27)	0.924	11 (8–18)	0.193	33 (24–42.5)	0.816
		Specialized secondary school (nonhealth)	20 (15–28)		13 (10–19)		34 (26–45.5)	
		Specialized secondary school (Healthcare)	21 (16–28)		12 (8–17)		34 (25–43)	
	Highest obtained degree	Secondary school	21 (15–28)	0.480	11 (8–17)	**0.003**	33 (24–42.75)	**0.046**
		Healthcare college (VOŠ)	19.5 (17–29.25)		17.5 (10.75–23.25)		38.5 (28.75–49.25)	
		University degree program	24 (17–28)		13 (9–22)		38 (28–47)	
		Correlation (*Spearman's rho*)	0.051	0.227	0.138	**0.001**	0.103	**0.014**
	Study program	Bachelor's degree program (Bc.)	21 (15–28)	0.947	12 (8–17)	0.593	34 (24–43)	0.641
		Master's degree program (Mgr.)	22 (14.5–27)		12 (8–19)		38 (29–40)	
	Study year	First year	18 (12–25)	**<** **0.001**	10 (7–16.75)	**0.002**	28.5 (20.25–40)	**<** **0.001**
		Second year	22 (16–28)		12 (8–18)		34 (26.5–45.5)	
		Third year	22 (16–28.5)		12 (9–17)		35 (26–43)	
		Fourth year	24 (21.5–30)		21 (15.5–26.5)		45 (37–58)	
		Correlation (*Spearman's rho*)	0.190	< 0.001	0.123	**0.004**	0.166	**<** **0.001**
	Study form	Full time	21 (15–27)	**0.032**	11 (8–15)	**<** **0.001**	32 (24–41)	**<** **0.001**
		Part time	24 (16–30)		16 (10–23)		39 (26–50)	

Employment	Employment status	Employed^ꝉ^	24 (16–29)	**0.006**	13 (8–20)	**<** **0.001**	36 (26–46)	**<** **0.001**
		Unemployed	20 (14.25–27)		11 (8–15)		31 (24–40)	
	^ꝉ^Workload	Part time (≤ 20 h per week)	22 (16–28)	0.682	12 (8–17)	**0.021**	34 (25–44)	0.198
		Part time (> 20 h per week)	23 (17–29)		13 (8–19)		36 (26–44)	
		Full time (40 h per week)	24 (16–30)		16 (10–21.75)		39.5 (28–50)	
	^ꝉ^Workplace	Intensive care unit (ICU)	25 (20–31)	**0.010**	15 (8–20)	0.739	40 (30–49)	0.122
		Standard care department	23 (16–28)		13 (9–18.5)		34 (26–46)	
		Other	20 (14–27)		13 (10–21)		35 (23–43)	

Prc	Self-perceived health	Correlation (*Spearman's rho*)	−0.302	**<** **0.001**	−0.262	**<** **0.001**	−0.314	**<** **0.001**
	Perceived exhaustion	Correlation (*Spearman's rho*)	0.436	**<** **0.001**	0.358	**<** **0.001**	0.439	**<** **0.001**

Musculoskeletal pain	Lower back pain	Yes	22 (16–28)	**0.006**	12 (9–18)	**<** **0.001**	34 (26–44)	**<** **0.001**
		No	18 (13.25–25)		10 (6–13)		28 (21.25–39.5)	
	Upper back pain	Yes	24 (17–29.75)	**<** **0.001**	14 (9.25–20)	**<** **0.001**	38 (28.25–48)	**<** **0.001**
		No	19 (13–25)		10 (7–15)		31 (22–40)	
	Shoulder region pain	Yes	24 (17–29)	**<** **0.001**	13 (9–19)	**<** **0.001**	37 (27–46)	**<** **0.001**
		No	19 (13–25)		10 (7–15)		30 (22.5–39)	
	Neck region pain	Yes	23 (17–29)	**<** **0.001**	12 (9–18)	**<** **0.001**	36 (27–46)	**<** **0.001**
		No	18 (12–25)		10 (7–14)		28 (21–38)	

Sleep quality	Enough sleep/work	Enough	17 (11.25–22)	**<** **0.001**	10 (6.25–14)	**<** **0.001**	27 (19–37)	**<** **0.001**
		Not enough	24 (17–29)		13 (9–19)		36 (27–47)	
	Enough sleep/rest	Enough	20 (15–26)	**<** **0.001**	11 (7–15)	**<** **0.001**	32 (23–40)	**<** **0.001**
		Not enough	25 (18–30)		15 (10–23)		40 (28–52)	
	Sleep quality/work	High quality	17 (11–24)	**<** **0.001**	10 (6–15)	**<** **0.001**	27 (19–37)	**<** **0.001**
		Low quality	26 (20–30)		14 (10–20.5)		40 (32–49.5)	
	Sleep quality/rest	High quality	20 (14–26)	**<** **0.001**	11 (7–15)	**<** **0.001**	32 (23–40)	**<** **0.001**
		Low quality	28 (20–32)		17 (11–24)		46 (32–54)	

Challenging experiences	Death	Yes	21 (16–28)	**0.033**	12 (8–17)	0.164	34 (25–43)	**0.047**
		No	18 (13–27)		10 (7.5–18.5)		28 (21–38.5)	
	Human suffering	Yes	21 (15–28)	0.219	12 (8–17)	0.234	34 (25–43)	0.197
		No	17 (11–24.5)		9 (6.5–15.5)		27 (17.5–37.5)	
	Aggressive patients	Yes	21 (16–28)	**0.004**	12 (8–17)	**0.008**	34 (25–44)	**0.002**
		No	17 (11–21.75)		9 (7–11.5)		27.5 (20–33.75)	
	Problematic patients	Yes	21 (15–28)	**0.005**	12 (8–17)	0.126	34 (25–43)	**0.021**
		No	10 (8–11.5)		6 (5–10.5)		16 (13–22)	
	Bullying	Yes	25 (18–30)	**<** **0.001**	14 (10–20)	**<** **0.001**	38.5 (30–48.25)	**<** **0.001**
		No	18 (13–25)		10 (7–15)		30 (22–40)	
	Discrimination	Yes	25 (18–30)	**<** **0.001**	14.5 (10–20)	**<** **0.001**	40 (29.75–48)	**<** **0.001**
		No	20 (15–27)		11 (8–16.5)		32 (23–42)	

*Note:* Mann–Whitney test (*U*), Kruskal–Wallis test (*H*), and nonparametric correlation (Spearman's *rho*) were conducted with a significance level (*Sig*.) < 0.05. Bold values represent statistically significant values (values below *p* 0.05).

^ꝉ^The employed subgroup.

**Table 5:** Training feedback and career intentions of Czech nursing students participating in the SYRI survey, August–November 2023 (*N* = 559).

Construct	Item	Outcome	Frequency (%)	Median (IQR)
Training expectations alignment	My clinical practice meets my expectations of working in healthcare.	10-point Likert scale		5 (3–8)
	My clinical practice matches and complements what I learned during my studies.	10-point Likert scale		6 (3–8)
	Overall	Composite score (Item I + II)		11 (7–15)

Endurance in nursing training	I expect to stay in healthcare after graduating.	10-point Likert scale		9 (6–10)
	I expect to successfully complete my current study program.	10-point Likert scale		9 (7–10)
	I am considering dropping out of my current nursing study program.	10-point Likert scale^(R)^		10 (8–10)
	Overall	Composite score (Item III + IV + V)		27 (21–30)

Career intentions	Increasing qualifications in nursing	Yes	466 (87.9%)	
		No	64 (12.1%)	
		5-point Likert scale (Never ∼ Daily)		3 (2–4)
	Increasing qualifications outside of nursing	Yes	327 (61.6%)	
		No	204 (38.4%)	
		5-point Likert scale (Never ∼ Daily)		2 (1–3)
	Quitting/giving up nursing profession	Yes	304 (55.6%)	
		No	243 (44.4%)	
		5-point Likert scale (Never ∼ Daily)		2 (1–3)
	Starting a different type of qualification/profession	Yes	268 (48.9%)	
		No	280 (51.1%)	
		5-point Likert scale (Never ∼ Daily)		1 (1–2)

*Note:* (R) is a reversed rating item.

**Table 6:** Training feedback of Czech nursing students participating in the SYRI survey, stratified by sociodemographic and anamnestic characteristics, August–November 2023 (*N* = 559).

	Variable	Outcome	Training expectations alignment: median (IQR)	Sig.	Endurance in nursing training: median (IQR)	Sig.
Sociodemographic	Gender	Female	11 (7–15)	0.380	27 (21–29.75)	0.928
		Male	12 (7–16.25)		26.5 (21–30)	
		Diverse	8 (3–9)		26 (12–26)	
	Age group	≤ 21 year old	12 (7.25–15)	**0.048**	26 (21–29)	0.195
		> 21 year old	10 (6–15)		27 (21–30)	
		Correlation (*Spearman's rho*)	−0.090	**0.034**	0.057	0.181
	BMI level	Underweight	11 (7.25–15)	0.906	27 (22.25–29.75)	0.838
		Normal weight	11 (7–15)		26.5 (21–29.25)	
		Overweight	12 (7–16)		27 (21.25–30)	
		Obesity	11 (6–15)		26 (20–30)	
		Correlation (*Spearman's rho*)	0.003	0.935	−0.017	0.698

Education	Secondary school	General secondary school	12 (7–16)	0.492	27 (22.5–30)	0.290
		Specialized secondary School (nonhealth)	13 (7–15.5)		27 (22–29.5)	
		Specialized secondary school (healthcare)	11 (7–15)		26 (21–29)	
	Highest obtained degree	Secondary school	11 (7–15)	**0.031**	26 (21–29)	**0.021**
		Healthcare college (VOŠ)	6 (4–13)		24 (18.25–30)	
		University degree program	13 (9–16)		29 (25–30)	
		Correlation (*Spearman's rho*)	−0.014	0.748	0.073	0.084
	Study program	Bachelor's degree program (Bc.)	11 (7–15)	0.522	26 (21–29)	**0.032**
		Master's degree program (Mgr.)	11 (9–15.5)		30 (28–30)	
	Study year	First year	12.5 (8.25–16)	**<** **0.001**	27 (21–30)	0.357
		Second year	11 (6–15)		26 (20–29)	
		Third year	10 (6–14)		27 (22–30)	
		Fourth year	5 (2.5–10)		22 (20.5–27.5)	
		Correlation (*Spearman's rho*)	−0.168	**<** **0.001**	0.033	0.434
	Study form	Full time	11 (7–15)	0.157	26 (21–29)	0.115
		Part time	12 (6–17)		27 (22–30)	

Employment	Employment status	Employed^ꝉ^	11 (6–15)	0.580	27 (22–30)	**0.001**
		Unemployed	12 (7–15)		25 (20–29)	
	^ꝉ^Workload	Part time (≤ 20 h per week)	11 (6–15)	0.112	28 (22–30)	0.457
		Part time (> 20 h per week)	11 (6–14)		26 (21–29)	
		Full time (40 h per week)	11.5 (8–17)		28 (22–30)	
	^ꝉ^Workplace	Intensive care unit (ICU)	9 (5–14)	0.158	29 (23–30)	0.596
		Standard care department	11 (7–15)		27 (22–30)	
		Other	12 (6–17)		28 (23–30)	

Prc	Self perceived health	Correlation (*Spearman's rho*)	0.254	**<** **0.001**	0.274	**<** **0.001**
	Perceived exhaustion	Correlation (*Spearman's rho*)	−0.298	**<** **0.001**	−0.358	**<** **0.001**

Musculoskeletal pain	Lower back pain	Yes	11 (6.75–15)	**0.029**	27 (21–29.25)	0.606
		No	13 (9–16)		27 (22–30)	
	Upper back pain	Yes	12 (7–16)	**0.022**	27 (22–30)	**0.003**
		No	10 (6.25–15)		26 (20–29)	
	Shoulder region pain	Yes	10 (6–15)	**0.009**	26 (20–29)	**0.002**
		No	12 (7–16)		27 (22–30)	
	Neck region pain	Yes	11 (6–15)	**0.013**	26 (21–29)	**0.015**
		No	13 (8–16)		27 (22–30)	

Sleep quality	Enough sleep/work	Enough	13 (9–16)	**<** **0.001**	27 (22–30)	**0.033**
		Not enough	10 (6–15)		26 (21–29)	
	Enough sleep/rest	Enough	12 (7–16)	**<** **0.001**	27 (22–30)	**<** **0.001**
		Not enough	10 (6–14)		24.5 (19–29)	
	Sleep quality/work	High quality	13 (10–16)	**<** **0.001**	28 (23–30)	**<** **0.001**
		Low quality	9 (5–14)		25 (20–29)	
	Sleep quality/rest	High quality	12 (7–16)	**0.002**	27 (22–30)	**<** **0.001**
		Low quality	9 (5–14)		22 (17–27)	

Challenging experiences	Death	Yes	12 (7–15)	0.195	26 (21–30)	0.959
		No	10 (7–14.5)		28 (19.5–29.5)	
	Human suffering	Yes	11 (7–15)	0.117	27 (21–30)	0.235
		No	8 (6–12)		25 (16.5–28.5)	
	Aggressive patients	Yes	11 (7–15)	0.093	26 (21–29)	**0.049**
		No	14 (8–16)		28 (22.25–30)	
	Problematic patients	Yes	11 (7–15)	0.713	27 (21–30)	0.980
		No	10 (7–13.25)		26.5 (22.25–28.5)	
	Bullying	Yes	9 (6–14)	**<** **0.001**	25 (19–29)	**<** **0.001**
		No	13 (9–16)		27 (23–30)	
	Discrimination	Yes	10 (6–15)	**0.020**	26 (18–29.25)	**0.032**
		No	12 (7–16)		27 (22–30)	

*Note:* Mann–Whitney test (*U*), Kruskal–Wallis test (*H*), and nonparametric correlation (Spearman's rho) were conducted with a significance level (Sig.) < 0.05. Bold values represent statistically significant values (values below *p* 0.05).

^ꝉ^The employed subgroup.

**Table 7:** Career intentions of Czech nursing students participating in the SYRI survey, stratified by sociodemographic and anamnestic characteristics, August–November 2023 (*N* = 559).

	**Variable**	**Outcome**	**Increasing qualifications in nursing**			**Increasing qualifications outside of nursing**		
			**No**	**Yes**	**Sig.**	**No**	**Yes**	**Sig.**

*Sociodemographic*	Gender	Female	56 (11.5%)	432 (88.5%)	0.084	190 (38.9%)	298 (61.1%)	0.247
		Male	6 (16.2%)	31 (83.8%)		14 (36.8%)	24 (63.2%)	
		Diverse	2 (40%)	3 (60%)		0 (0.0%)	5 (100.0%)	
	Age group	≤ 21 years old	39 (12.6%)	270 (87.4%)	0.662	114 (37.3%)	192 (62.7%)	0.494
		> 21 years old	25 (11.4%)	195 (88.6%)		90 (40.2%)	134 (59.8%)	
		Correlation (*Spearman's rho*)	−0.017		0.691	0.005		0.907
	BMI level	Underweight	4 (11.8%)	30 (88.2%)	0.550	10 (31.3%)	22 (68.8%)	0.329
		Normal weight	39 (13.4%)	253 (86.6%)		113 (38.0%)	184 (62.0%)	
		Overweight	16 (12.4%)	113 (87.6%)		55 (43.7%)	71 (56.3%)	
		Obesity	5 (7.0%)	66 (93.0%)		23 (31.9%)	49 (68.1%)	
		Correlation (*Spearman's rho*)	0.027		0.537	0.009		0.841

Education	Secondary school	General secondary school	13 (13.3%)	85 (86.7%)	0.766	36 (36.7%)	62 (63.3%)	0.769
		Specialized secondary school (nonhealth)	8 (14.3%)	48 (85.7%)		20 (35.1%)	37 (64.9%)	
		Specialized secondary school (healthcare)	43 (11.4%)	333 (88.6%)		148 (39.4%)	228 (60.6%)	
	Highest obtained degree	Secondary school	55 (11.7%)	417 (88.3%)	0.519	179 (38.1%)	291 (61.9%)	0.557
		Healthcare college (VOŠ)	3 (13.0%)	20 (87.0%)		8 (33.3%)	16 (66.7%)	
		University degree program	6 (17.1%)	29 (82.9%)		17 (45.9%)	20 (54.1%)	
		Correlation (*Spearman's rho*)	0.008		0.858	0.023		0.601
	Study program	Bachelor's degree program (Bc.)	62 (12.0%)	455 (88.0%)	0.663	195 (37.8%)	321 (62.2%)	0.081
		Master's degree program (Mgr.)	2 (15.4%)	11 (84.6%)		9 (60.0%)	6 (40.0%)	
	Study year	First year	14 (10.1%)	125 (89.9%)	0.247	57 (41.3%)	81 (58.7%)	0.481
		Second year	18 (10.0%)	162 (90.0%)		62 (34.3%)	119 (65.7%)	
		Third year	32 (15.7%)	172 (84.3%)		83 (40.5%)	122 (59.5%)	
		Fourth year	0 (0.0%)	7 (100.0%)		2 (28.6%)	5 (71.4%)	
		Correlation (*Spearman's rho*)	−0.133		**0.002**	−0.018		0.675
	Study form	Full time	56 (14.7%)	326 (85.3%)	**0.003**	147 (38.6%)	234 (61.4%)	0.901
		Part–time	8 (5.4%)	140 (94.6%)		57 (38.0%)	93 (62.0%)	

Employment	Employment status	Unemployed	41 (14.6%)	240 (85.4%)	0.059	106 (37.7%)	175 (62.3%)	0.727
		Employed^ꝉ^	23 (9.2%)	226 (90.8%)		98 (39.2%)	152 (60.8%)	
	^ꝉ^Workload	Part time (≤ 20 h per week)	12 (11.0%)	97 (89.0%)	0.641	44 (41.1%)	63 (58.9%)	0.300
		Part time (> 20 h per week)	5 (9.1%)	50 (90.9%)		17 (30.4%)	39 (69.6%)	
		Full time (40 h per week)	6 (7.1%)	79 (92.9%)		37 (42.5%)	50 (57.5%)	
	^ꝉ^Workplace	Intensive care unit (ICU)	3 (5.5%)	52 (94.5%)	0.388	25 (45.5%)	30 (54.5%)	0.235
		Standard care department	18 (11.5%)	138 (88.5%)		62 (39.7%)	94 (60.3%)	
		Other	2 (5.3%)	36 (94.7%)		11 (28.2%)	28 (71.8%)	

Prc	Self-perceived health	Correlation (*Spearman's rho*)	0.070		0.105	−0.140		**0.001**
	Perceived exhaustion	Correlation (*Spearman's rho*)	−0.011		0.793	0.188		**<** **0.001**

Musculoskeletal pain	Lower back pain	No	16 (17.0%)	78 (83.0%)	0.081	46 (48.9%)	48 (51.1%)	**0.027**
		Yes	45 (10.6%)	379 (89.4%)		156 (36.6%)	270 (63.4%)	
	Upper back pain	No	41 (14.9%)	234 (85.1%)	0.060	127 (45.7%)	151 (54.3%)	**0.003**
		Yes	21 (9.3%)	204 (90.7%)		73 (32.7%)	150 (67.3%)	
	Shoulder region pain	No	30 (12.6%)	209 (87.4%)	0.585	107 (44.4%)	134 (55.6%)	**0.008**
		Yes	29 (11.0%)	235 (89.0%)		87 (33.0%)	177 (67.0%)	
	Neck region pain	No	21 (13.5%)	135 (86.5%)	0.468	70 (44.6%)	87 (55.4%)	0.076
		Yes	40 (11.2%)	317 (88.8%)		130 (36.3%)	228 (63.7%)	

Sleep quality	Enough sleep/work	Not enough	39 (10.5%)	331 (89.5%)	0.099	133 (35.8%)	238 (64.2%)	0.064
		Enough	25 (15.6%)	135 (84.4%)		71 (44.4%)	89 (55.6%)	
	Enough sleep/rest	Not enough	24 (15.8%)	128 (84.2%)	0.096	50 (32.7%)	103 (67.3%)	0.084
		Enough	40 (10.6%)	338 (89.4%)		154 (40.7%)	224 (59.3%)	
	Sleep quality/work	Low quality	25 (10.8%)	206 (89.2%)	0.212	72 (31.0%)	160 (69.0%)	**<** **0.001**
		High quality	17 (15.6%)	92 (84.4%)		56 (50.9%)	54 (49.1%)	
	Sleep quality/rest	Low quality	14 (18.2%)	63 (81.8%)	0.060	25 (32.1%)	53 (67.9%)	0.205
		High quality	34 (10.5%)	291 (89.5%)		129 (39.8%)	195 (60.2%)	

Challenging experiences	Death	No	13 (23.6%)	42 (76.4%)	**0.005**	24 (40.7%)	35 (59.3%)	0.705
		Yes	51 (10.7%)	424 (89.3%)		180 (38.1%)	292 (61.9%)	
	Human suffering	No	4 (40.0%)	6 (60.0%)	**0.023**	7 (63.6%)	4 (36.4%)	0.116
		Yes	60 (11.5%)	460 (88.5%)		197 (37.9%)	323 (62.1%)	
	Aggressive patients	No	6 (20.0%)	24 (80.0%)	0.241	17 (53.1%)	15 (46.9%)	0.078
		Yes	58 (11.6%)	442 (88.4%)		187 (37.5%)	312 (62.5%)	
	Problematic patients	No	2 (66.7%)	1 (33.3%)	**0.040**	1 (25.0%)	3 (75.0%)	1.000
		Yes	62 (11.8%)	465 (88.2%)		203 (38.5%)	324 (61.5%)	
	Bullying	No	34 (11.1%)	272 (88.9%)	0.426	120 (39.5%)	184 (60.5%)	0.563
		Yes	30 (13.4%)	194 (86.6%)		84 (37.0%)	143 (63.0%)	
	Discrimination	No	56 (13.6%)	355 (86.4%)	**0.042**	170 (41.5%)	240 (58.5%)	**0.008**
		Yes	8 (6.7%)	111 (93.3%)		34 (28.1%)	87 (71.9%)	

	**Variable**	**Outcome**	**Quitting/giving up nursing profession**			**Starting a different type of qualification/profession**		
			**No**	**Yes**	**Sig.**	**No**	**Yes**	**Sig.**

Sociodemographic	Gender	Female	220 (43.7%)	283 (56.3%)	0.376	262 (51.9%)	243 (48.1%)	0.101
		Male	20 (50.0%)	20 (50.0%)		18 (46.2%)	21 (53.8%)	
		Diverse	3 (75.0%)	1 (25.0%)		0 (0.0%)	4 (100.0%)	
	Age group	≤ 21 years old	134 (41.7%)	187 (58.3%)	0.148	161 (50.0%)	161 (50.0%)	0.506
		> 21 years old	108 (48.0%)	117 (52.0%)		119 (52.9%)	106 (47.1%)	
		Correlation (*Spearman's rho*)	−0.025		0.568	−0.002		0.960
	BMI level	Underweight	10 (31.3%)	22 (68.8%)	0.364	12 (40.0%)	18 (60.0%)	**0.032**
		Normal weight	139 (45.1%)	169 (54.9%)		155 (50.3%)	153 (49.7%)	
		Overweight	61 (47.3%)	68 (52.7%)		80 (60.6%)	52 (39.4%)	
		Obesity	30 (40.5%)	44 (59.5%)		31 (41.9%)	43 (58.1%)	
		Correlation (*Spearman's rho*)	0.011		0.805	−0.010		0.809

Education	Secondary school	General secondary school	46 (46.9%)	52 (53.1%)	0.596	46 (46.9%)	52 (53.1%)	0.421
		Specialized secondary school (nonhealth)	28 (49.1%)	29 (50.9%)		33 (57.9%)	24 (42.1%)	
		Specialized secondary school (healthcare)	169 (43.1%)	223 (56.9%)		201 (51.1%)	192 (48.9%)	
	Highest obtained degree	Secondary school	210 (43.0%)	278 (57.0%)	0.152	251 (51.2%)	239 (48.8%)	0.950
		Healthcare college (VOŠ)	12 (52.2%)	11 (47.8%)		11 (47.8%)	12 (52.2%)	
		University degree program	21 (58.3%)	15 (41.7%)		18 (51.4%)	17 (48.6%)	
		Correlation (*Spearman's rho*)	−0.068		0.111	0.025		0.558
	Study program	Bachelor's degree program (Bc.)	234 (44.0%)	298 (56.0%)	0.218	270 (50.7%)	263 (49.3%)	0.221
		Master's degree program (Mgr.)	9 (60.0%)	6 (40.0%)		10 (66.7%)	5 (33.3%)	
	Study year	First year	77 (53.8%)	66 (46.2%)	**0.034**	90 (62.9%)	53 (37.1%)	**0.005**
		Second year	76 (40.2%)	113 (59.8%)		94 (50.0%)	94 (50.0%)	
		Third year	89 (42.6%)	120 (57.4%)		94 (44.8%)	116 (55.2%)	
		Fourth year	1 (16.7%)	5 (83.3%)		2 (28.6%)	5 (71.4%)	
		Correlation (*Spearman's rho*)	0.055		0.200	0.116		**0.007**
	Study form	Full time	166 (42.0%)	229 (58.0%)	0.069	191 (48.2%)	205 (51.8%)	**0.030**
		Part time	77 (50.7%)	75 (49.3%)		89 (58.6%)	63 (41.4%)	

Employment	Employment status	Unemployed	123 (42.3%)	168 (57.7%)	0.279	132 (45.4%)	159 (54.6%)	**0.004**
		Employed^ꝉ^	120 (46.9%)	136 (53.1%)		148 (57.6%)	109 (42.4%)	
	^ꝉ^Workload	Part time (≤ 20 h per week)	50 (45.5%)	60 (54.5%)	0.391	67 (60.9%)	43 (39.1%)	0.328
		Part time (> 20 h per week)	23 (41.1%)	33 (58.9%)		28 (49.1%)	29 (50.9%)	
		Full time (40 h per week)	47 (52.2%)	43 (47.8%)		53 (58.9%)	37 (41.1%)	
	^ꝉ^Workplace	Intensive care unit (ICU)	25 (45.5%)	30 (54.5%)	0.949	31 (55.4%)	25 (44.6%)	0.699
		Standard care department	75 (46.9%)	85 (53.1%)		91 (56.9%)	69 (43.1%)	
		Other	20 (48.8%)	21 (51.2%)		26 (63.4%)	15 (36.6%)	

Prc	Self-perceived health	Correlation (*Spearman's rho*)	−0.239		**<** **0.001**	−0.225		**<** **0.001**
	Perceived exhaustion	Correlation (*Spearman's rho*)	0.444		**<** **0.001**	0.352		**<** **0.001**

Musculoskeletal pain	Lower back pain	No	54 (53.5%)	47 (46.5%)	**0.041**	54 (54.0%)	46 (46.0%)	0.537
		Yes	183 (42.3%)	250 (57.7%)		220 (50.6%)	215 (49.4%)	
	Upper back pain	No	145 (50.3%)	143 (49.7%)	**0.004**	165 (57.7%)	121 (42.3%)	**<** **0.001**
		Yes	87 (37.8%)	143 (62.2%)		100 (43.1%)	132 (56.9%)	
	Shoulder region pain	No	123 (48.4%)	131 (51.6%)	**0.023**	135 (53.6%)	117 (46.4%)	0.147
		Yes	103 (38.6%)	164 (61.4%)		127 (47.2%)	142 (52.8%)	
	Neck region pain	No	92 (54.8%)	76 (45.2%)	**<** **0.001**	89 (53.3%)	78 (46.7%)	0.463
		Yes	142 (39.3%)	219 (60.7%)		181 (49.9%)	182 (50.1%)	

Sleep quality	Enough sleep/work	Not enough	142 (37.4%)	238 (62.6%)	**<** **0.001**	182 (48.0%)	197 (52.0%)	**0.031**
		Enough	101 (60.5%)	66 (39.5%)		98 (58.0%)	71 (42.0%)	
	Enough sleep/rest	Not enough	55 (34.6%)	104 (65.4%)	**0.003**	71 (44.7%)	88 (55.3%)	0.054
		Enough	188 (48.5%)	200 (51.5%)		209 (53.7%)	180 (46.3%)	
	Sleep quality/work	Low quality	75 (31.4%)	164 (68.6%)	**<** **0.001**	95 (39.7%)	144 (60.3%)	**<** **0.001**
		High quality	73 (64.0%)	41 (36.0%)		76 (66.1%)	39 (33.9%)	
	Sleep quality/rest	Low quality	27 (33.8%)	53 (66.3%)	**0.014**	34 (43.0%)	45 (57.0%)	0.155
		High quality	164 (49.0%)	171 (51.0%)		174 (51.9%)	161 (48.1%)	

Challenging experiences	Death	No	29 (48.3%)	31 (51.7%)	0.518	31 (51.7%)	29 (48.3%)	0.925
		Yes	214 (43.9%)	273 (56.1%)		249 (51.0%)	239 (49.0%)	
	Human suffering	No	6 (54.5%)	5 (45.5%)	0.550	6 (54.5%)	5 (45.5%)	0.817
		Yes	237 (44.2%)	299 (55.8%)		274 (51.0%)	263 (49.0%)	
	Aggressive patients	No	21 (61.8%)	13 (38.2%)	**0.036**	19 (57.6%)	14 (42.4%)	0.442
		Yes	222 (43.3%)	291 (56.7%)		261 (50.7%)	254 (49.3%)	
	Problematic patients	No	3 (75.0%)	1 (25.0%)	0.328	3 (75.0%)	1 (25.0%)	0.624
		Yes	240 (44.2%)	303 (55.8%)		277 (50.9%)	267 (49.1%)	
	Bullying	No	165 (52.2%)	151 (47.8%)	**<** **0.001**	185 (58.2%)	133 (41.8%)	**<** **0.001**
		Yes	78 (33.8%)	153 (66.2%)		95 (41.3%)	135 (58.7%)	
	Discrimination	No	193 (46.0%)	227 (54.0%)	0.191	225 (53.3%)	197 (46.7%)	**0.057**
		Yes	50 (39.4%)	77 (60.6%)		55 (43.7%)	71 (56.3%)	

*Note:* Chi-squared test (*χ*^2^), Fisher's exact test, and nonparametric correlation (Spearman's *rho*) were conducted with a significance level (*Sig*.) < 0.05. Bold values represent statistically significant values (values below *p* 0.05).

^ꝉ^The employed subgroup.

**Table 8:** Correlates of training feedback and career intentions of Czech nursing students participating in the SYRI survey, August–November 2023 (*N* = 559).

Correlate	Training expectations alignment	Endurance in nursing training	Increasing of nursing qualifications	Increasing of nonnursing qualif.	Quitting of nursing profession	Starting different profession
Job demand	−0.157 (**<** **0.001**)	−0.192 (**< 0.001**)	0.087 (**0.046**)	0.148 (**0.001**)	0.215 (**<** **0.001**)	0.141 (**0.001**)
Job control	0.207 (**<** **0.001**)	0.208 (**< 0.001**)	0.001 (0.979)	−0.076 (0.081)	−0.231 (**<** **0.001**)	−0.182 (**<** **0.001**)
Social support	0.634 (**<** **0.001**)	0.325 (**<** **0.001**)	0.182 (**<** **0.001**)	−0.121 (**0.005**)	−0.357 (**<** **0.001**)	−0.355 (**<** **0.001**)
Work-to-family conflict (WFC)	−0.330 (**<** **0.001**)	−0.214 (**<** **0.001**)	0.041 (0.348)	0.143 (**0.001**)	0.326 (**<** **0.001**)	0.270 (**<** **0.001**)
Family-to-work conflict (FWC)	−0.254 (**<** **0.001**)	−0.254 (**<** **0.001**)	0.059 (0.177)	0.154 (**<** **0.001**)	0.269 (**<** **0.001**)	0.191 (**<** **0.001**)
Work and family conflict scale (WAFCS)	−0.327 (**<** **0.001**)	−0.255 (**<** **0.001**)	0.051 (0.238)	0.157 (**<** **0.001**)	0.332 (**<** **0.001**)	0.255 (**<** **0.001**)
Work ability index (WAI)	0.309 (**<** **0.001**)	0.378 (**<** **0.001**)	0.110 (**0.011**)	−0.170 (**<** **0.001**)	−0.339 (**<** **0.001**)	−0.329 (**<** **0.001**)

*Note:* Correlation's *rho* (*Sig*. value). Bold values refer to statistically significant values (*p* <0.05).

**Table 9:** Multilinear regression (MLR) models of training feedback of Czech nursing students participating in the SYRI survey, August–November 2023 (*N* = 559).

Training expectations alignment	Adjusted *R*-squared = 39%		Endurance in nursing training	Adjusted *R*-squared = 23.4%	
	AOR (95% CI)	Sig.		AOR (95% CI)	Sig.
Self-perceived health	−0.111 (−0.324–0.102)	0.306	Self-perceived health	−0.079 (−0.315–0.158)	0.514
Perceived exhaustion	−0.094 (−0.295–0.106)	0.354	Perceived exhaustion	−0.247 (−0.470–−0.025)	**0.030**
Job demand	−0.162 (−0.435–0.112)	0.245	Job demand	−0.109 (−0.413–0.195)	0.481
Job control	−0.113 (−0.366–0.141)	0.382	Job control	−0.106 (−0.387–0.175)	0.459
Social support	1.021 (0.826–1.216)	**<** **0.001**	Social support	0.191 (−0.026–0.407)	0.084
Work-to-family conflict (WFC)	−0.043 (−0.134–0.049)	0.358	Work-to-family conflict (WFC)	0.062 (−0.039–0.164)	0.227
Family-to-work conflict (FWC)	−0.054 (−0.156–0.048)	0.296	Family-to-work conflict (FWC)	−0.133 (−0.246–−0.020)	**0.021**
Work ability index (WAI)	−0.058 (−0.196–0.079)	0.404	Work ability index (WAI)	0.309 (0.157–0.462)	**<** **0.001**

*Note:* All models were adjusted for gender, age, BMI, secondary school type, highest obtained degree, study program, study year, study form, workload, and workplace. Bold values refer to statistically significant values (*p* <0.05).

**Table 10:** Multivariable logistic regression models of career intentions of Czech nursing students participating in the SYRI survey, August–November 2023 (*N* = 559).

Increasing of nursing qualifications	Nagelkerke *R* square = 25.5%		Increasing of nonnursing qualifications	Nagelkerke *R* square = 12.8%	
	AOR (95% CI)	Sig.		AOR (95% CI)	Sig.
Self-perceived health	1.093 (0.884–1.350)	0.412	Self-perceived Health	1.029 (0.918–1.154)	0.623
Perceived exhaustion	1.038 (0.856–1.259)	0.702	Perceived exhaustion	1.145 (1.023–1.283)	**0.019**
Job demand	1.018 (0.787–1.317)	0.892	Job demand	0.960 (0.830–1.112)	0.589
Job control	1.029 (0.817–1.297)	0.806	Job control	1.121 (0.977–1.286)	0.104
Social support	1.308 (1.087–1.573)	**0.004**	Social support	1.013 (0.914–1.122)	0.809
Work-to-family conflict (WFC)	1.104 (1.019–1.197)	**0.015**	Work-to-family conflict (WFC)	1.005 (0.958–1.054)	0.843
Family-to-work conflict (FWC)	0.974 (0.886–1.071)	0.588	Family-to-ork conflict (FWC)	0.995 (0.942–1.052)	0.863
Work ability index (WAI)	1.015 (0.897–1.148)	0.815	Work ability index (WAI)	0.949 (0.881–1.023)	0.169

**Quitting of nursing profession**	**Nagelkerke *R* square = 22.9%**		**Starting a different study/profession**	**Nagelkerke *R* square = 22.5%**	
	**AOR (95% CI)**	**Sig.**		**AOR (95% CI)**	**Sig.**

Self-perceived health	1.067 (0.948–1.201)	0.282	Self-perceived health	0.998 (0.891–1.118)	0.974
Perceived exhaustion	1.139 (1.019–1.273)	**0.022**	Perceived exhaustion	1.076 (0.967–1.198)	0.179
Job demand	1.075 (0.925–1.248)	0.345	Job demand	0.913 (0.784–1.064)	0.243
Job control	1.076 (0.938–1.234)	0.295	Job control	1.073 (0.935–1.231)	0.318
Social support	0.904 (0.812–1.005)	0.061	Social support	0.957 (0.860–1.064)	0.417
Work-to-family conflict (WFC)	1.038 (0.989–1.089)	0.132	Work-to-family conflict (WFC)	1.023 (0.974–1.076)	0.365
Family-to-work conflict (FWC)	0.987 (0.934–1.043)	0.642	Family-to-work conflict (FWC)	1.017 (0.963–1.074)	0.549
Work ability index (WAI)	0.908 (0.839–0.982)	**0.016**	Work ability index (WAI)	0.891 (0.825–0.963)	**0.003**

*Note:* All models were adjusted for gender, age, BMI, secondary school type, highest obtained degree, study program, study year, study form, workload, and workplace. Bold values represent statistically significant values (values below *p* 0.05).

## Data Availability

The data supporting this study's findings are available on request from the corresponding author. The data are not publicly available due to privacy or ethical restrictions.

## Supporting Information

Checklist of items that should be included in reports of cohort studies.

## References

[1] Canzan F., Saiani L., Mezzalira E., Allegrini E., Caliaro A., Ambrosi E. (2022). Why Do Nursing Students Leave Bachelor Program? Findings From a Qualitative Descriptive Study. *BMC Nursing*.

[2] Visiers-Jiménez L., Kuokkanen L., Leino-Kilpi H. (2022). Graduating Nursing Students’ Empowerment and Related Factors: Comparative Study in Six European Countries. *Healthcare (Basel)*.

[3] Kim J., Chae D., Yoo J. Y. (2021). Reasons Behind Generation Z Nursing Students’ Intentions to Leave Their Profession: A Cross-Sectional Study. *Inquiry: The Journal of Health Care Organization, Provision, and Financing*.

[4] Hu H., Wang C., Lan Y., Wu X. (2022). Nurses’ Turnover Intention, Hope and Career Identity: The Mediating Role of Job Satisfaction. *BMC Nursing*.

[5] Koskinen S., Burke E., Fatkulina N. (2022). Graduating Nurse Students’ Interest in Older People Nursing-A Cross-Sectional Survey in Six European Countries. *International Journal of Older People Nursing*.

[6] Alkan E., Cushen-Brewster N., Anyanwu P. (2024). Investigating Healthcare Workforce Recruitment and Retention: A Mixed-Methods Study Protocol. *BMJ Open*.

[7] Hambridge K., Banerjee S., Winfield L., Gripton J. (2023). Origins, Characteristics and Destination of Nursing Students in South West England. *BMC Nursing*.

[8] Karasek R. (1979). *Job Demands, Control, and Support Model*.

[9] Häusser J. A., Mojzisch A., Niesel M., Schulz-Hardt S. (2010). Ten Years On: A Review of Recent Research on the Job Demand–Control (-Support) Model and Psychological Well-Being. *Work & Stress*.

[10] Luchman J. N., González-Morales M. G. (2013). Demands, Control, and Support: A Meta-Analytic Review of Work Characteristics Interrelationships. *Journal of Occupational Health Psychology*.

[11] Lyu X.-C., Huang S.-S., Ye X.-M., Zhang L.-Y., Zhang P., Wang Y.-J. (2024). What Influences Newly Graduated Registered Nurses’ Intention to Leave the Nursing Profession? An Integrative Review. *BMC Nursing*.

[12] Maier C. B., Köppen J., Kleine J., McHugh M. D., Sermeus W., Aiken L. H. (2023). Recruiting and Retaining Bachelor Qualified Nurses in German Hospitals (Bsn4hospital): Protocol of a Mixed-Methods Design. *BMJ Open*.

[13] Li L., Xu R., Wang S. (2024). Moderating Effect of Family Structure on the Relationship Between Early Clinical Exposure and Emotional Labor of Nursing Students: A Cross-Sectional Study. *BMC Nursing*.

[14] Bakker E. J. M., Kox J. H. A. M., Boot C. R. L., Francke A. L., van der Beek A. J., Roelofs P. D. D. M. (2020). Improving Mental Health of Student and Novice Nurses to Prevent Dropout: A Systematic Review. *Journal of Advanced Nursing*.

[15] Navajas-Romero V., Ariza-Montes A., Hernández-Perlines F. (2020). Analyzing the Job Demands-Control-Support Model in Work-Life Balance: A Study Among Nurses in the European Context. *International Journal of Environmental Research and Public Health*.

[16] Wong K. P., Zhang B., Xie Y. J. (2024). Impacts of Job Demands on Turnover Intention Among Registered Nurses in Hong Kong Public Hospitals: Exploring the Mediating Role of Burnout and Moderating Effect of Pay Level Satisfaction. *Journal of Nursing Management*.

[17] Asiedu E. E. A., Annor F., Amponsah-Tawiah K., Dartey-Baah K. (2018). Juggling Family and Professional Caring Role Demands, Work-Family Conflict and Burnout among Registered Nurses in Ghana. *Nursing Open*.

[18] Tóthová V., Sedláková G. (2008). Nursing Education in the Czech Republic. *Nurse Education Today*.

[19] Czech Statistical Office (CZSO) (2022). *Students and Graduates of Universities in the Czech Republic–2001–2021*.

[20] Hasselhorn H.-M., Mueller B. H., Tackenberg P. (2003). Investigating Premature Departure From the Nursing Professions in Europe–The European Next-Study. *Working Conditions and Intent to Leave the Profession Among Nursing Staff in Europe 9*.

[21] Hasselhorn H.-M., Hans Müller B., Tackenberg P. (2005). *Next Scientific Report July 2005*.

[22] Bakker E. J. M., Kox J. H. A. M., Miedema H. S. (2018). Physical and Mental Determinants of Dropout and Retention Among Nursing Students: Protocol of the Spring Cohort Study. *BMC Nursing*.

[23] Ilmarinen J. (2006). The Work Ability Index (WAI). *Occupational Medicine*.

[24] Haslam D., Filus A., Morawska A., Sanders M. R., Fletcher R. (2015). The Work–Family Conflict Scale (WAFCS): Development and Initial Validation of a Self-Report Measure of Work-Family Conflict for Use With Parents. *Child Psychiatry and Human Development*.

[25] Bakker E. J. M., Verhaegh K. J., Kox J. H. A. M. (2019). Late Dropout From Nursing Education: An Interview Study of Nursing Students’ Experiences and Reasons. *Nurse Education in Practice*.

[26] NHS England (2023). *NHS Long Term Workforce Plan*.

[27] Lin Y., Hu Z., Danaee M., Alias H., Wong Li P. (2021). The Impact of the COVID-19 Pandemic on Future Nursing Career Turnover Intention Among Nursing Students. *Risk Management and Healthcare Policy*.

[28] Alghtany S., Madhuvu A., Fooladi E., Crawford K. (2024). Assessment of Academic Burnout and Professional Self-Concept in Undergraduate Nursing Students: A Cross-Sectional Study. *Journal of Professional Nursing*.

[29] Wongkoey D. (2024). The Difference in Achievement Motivation Based on Planning, Participation, Diligence, Responsibility, and Endurance Among Asia Pacific University Students. *APHEIT International Journal of Interdisciplinary Social Sciences and Technology*.

[30] Connelly D. M., Hay M. E., Guitar N. A., Prentice K. (2024). Bridging Educational Grant in Nursing (BEGIN) Students’ Intentions for Retention in Long-Term, Home and Community Care: A Survey Protocol. *BMJ Open*.

[31] Al Sabei S. D., Al Harthy M. S., Al Harthy A. S., Al Harthy S. S., Al Harthy S. S. (2020). Nursing Work Environment, Turnover Intention, Job Burnout, and Perceived Quality of Care Among Nurses in Oman. *Journal of Nursing Management*.

[32] Pien L. C., Cheng W. J., Chou K. R., Lin L. C. (2021). Effect of Work-Family Conflict, Psychological Job Demand, and Job Control on the Health Status of Nurses. *International Journal of Environmental Research and Public Health*.

[33] Martinez M. C., Latorre M. D. R. D. D. O., Fischer F. M. (2021). Factors Associated With Work Ability and Intention to Leave Nursing Profession: A Nested Case-Control Study. *Industrial Health*.

[34] Zhang J., Shields L., Ma B. (2022). The Clinical Learning Environment, Supervision and Future Intention to Work as a Nurse in Nursing Students: A Cross-Sectional and Descriptive Study. *BMC Medical Education*.

[35] Lundell Rudberg S., Westerbotn M., Sormunen T., Scheja M., Lachmann H. (2022). Undergraduate Nursing Students’ Experiences of Becoming a Professional Nurse: A Longitudinal Study. *BMC Nursing*.

